# Microvascular destabilization and intricated network of the cytokines in diabetic retinopathy: from the perspective of cellular and molecular components

**DOI:** 10.1186/s13578-024-01269-7

**Published:** 2024-06-27

**Authors:** Xia Sheng, Chunmei Zhang, Jiwei Zhao, Jianping Xu, Peng Zhang, Quanju Ding, Jingfa Zhang

**Affiliations:** 1 People’s Hospital of Huangdao District, Qingdao, Shandong Province, China; 2 Department of Ophthalmology, Shanghai General Hospital (Shanghai First People’s Hospital), Shanghai Jiao Tong University School of Medicine, Shanghai, China; 3 Shanghai Key Laboratory of Ocular Fundus Diseases, Shanghai Engineering Center for Visual Science and Photomedicine, Shanghai Engineering Center for Precise Diagnosis and Treatment of Eye Diseases, National Clinical Research Center for Eye Diseases, Shanghai, China; 4 The International Eye Research Institute of The Chinese University of Hong Kong (Shenzhen), Shenzhen, China; 5 C-MER (Shenzhen) Dennis Lam Eye Hospital, Shenzhen, China; 6 C-MER International Eye Care Group, C-MER Dennis Lam & Partners Eye Center, Hong Kong, China

**Keywords:** Diabetic retinopathy, Inner blood-retinal barrier, Neurovascular unit, Microvascular destabilization, Endothelial cell, Pericyte

## Abstract

Microvascular destabilization is the primary cause of the inner blood-retinal barrier (iBRB) breakdown and increased vascular leakage in diabetic retinopathy (DR). Microvascular destabilization results from the combinational effects of increased levels of growth factors and cytokines, involvement of inflammation, and the changed cell-to-cell interactions, especially the loss of endothelial cells and pericytes, due to hyperglycemia and hypoxia. As the manifestation of microvascular destabilization, the fluid transports via paracellular and transcellular routes increase due to the disruption of endothelial intercellular junctional complexes and/or the altered caveolar transcellular transport across the retinal vascular endothelium. With diabetes progression, the functional and the structural changes of the iBRB components, including the cellular and noncellular components, further facilitate and aggravate microvascular destabilization, resulting in macular edema, the neuroretinal damage and the dysfunction of retinal inner neurovascular unit (iNVU). Although there have been considerable recent advances towards a better understanding of the complex cellular and molecular network underlying the microvascular destabilization, some still remain to be fully elucidated. Recent data indicate that targeting the intricate signaling pathways may allow to against the microvascular destabilization. Therefore, efforts have been made to better clarify the cellular and molecular mechanisms that are involved in the microvascular destabilization in DR. In this review, we discuss: (1) the brief introduction of DR and microvascular destabilization; (2) the cellular and molecular components of iBRB and iNVU, and the breakdown of iBRB; (3) the matrix and cell-to-cell contacts to maintain microvascular stabilization, including the endothelial glycocalyx, basement membrane, and various cell–cell interactions; (4) the molecular mechanisms mediated cell–cell contacts and vascular cell death; (5) the altered cytokines and signaling pathways as well as the intricate network of the cytokines involved in microvascular destabilization. This comprehensive review aimed to provide the insights for microvascular destabilization by targeting the key molecules or specific iBRB cells, thus restoring the function and structure of iBRB and iNVU, to treat DR.

## Introduction of diabetic retinopathy (DR) and microvascular destabilization

**Fig. 1:**
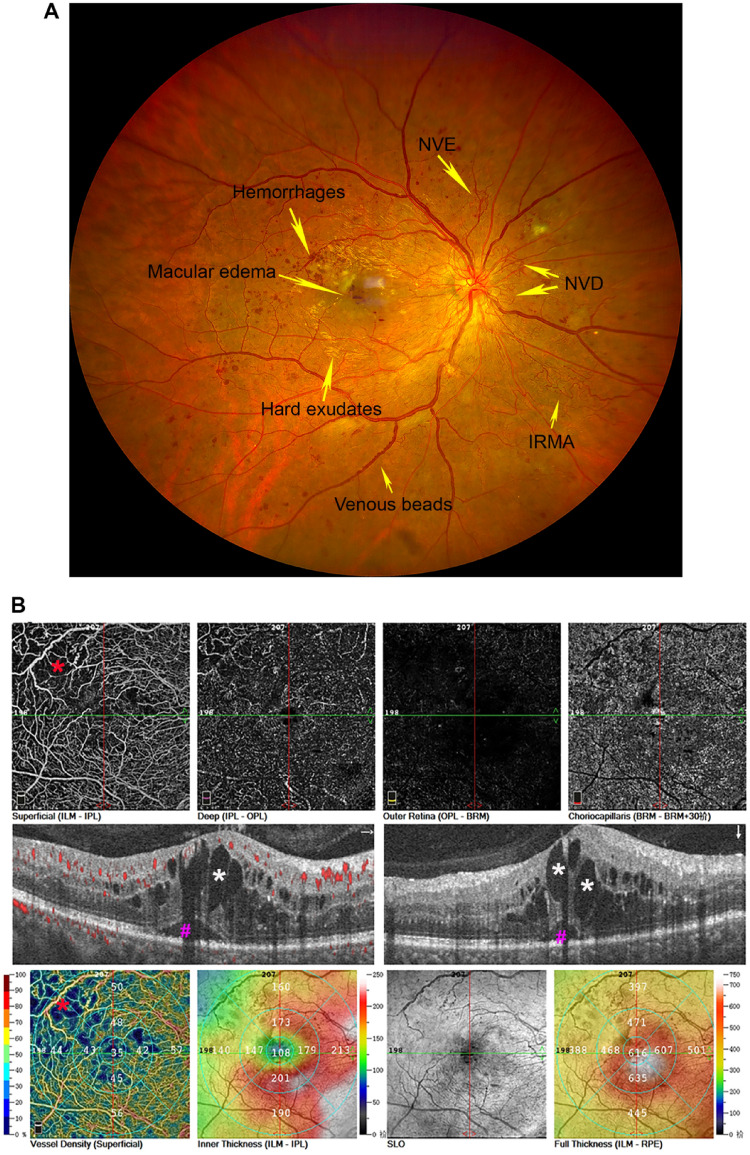
The representative figures of PDR and DME in a 31-year-old male, who was diagnosed with PDR and DME in his right eye. **A** The ultra-wide-field imaging of the fundus (CLARUS 500; Carl Zeiss, Dublin, USA) demonstrated the characteristic features of PDR in his right eye, which showed multiple microvascular abnormalities (indicated with yellow arrow), including hemorrhages, hard exudates, venous beads, IRMA, macular edema, NVD and NVE. **B** DME was evidenced on OCTA (RTVue XR Avanti OCT system, Optovue, Inc., Fremont, CA, USA). Mixed type of DME was seen on the B-scan of OCTA, demonstrating intraretinal fluid, cystoid edema (white *), and subretinal fluid (purple #) accumulation. The central subfield thickness was 616 µm on the lower right (**B**), showing significant macular edema. The capillary loss and non-perfusion area (indicated with red * on both upper left and lower left (**B**) was seen in the superficial capillary plexus of the superior temporal quadrant of the macula on OCTA. DME: diabetic macular edema; IRMA: intraretinal microvascular abnormalities; NVD: neovascularization on the optic disc; NVE: neovascularization elsewhere in the retina; OCTA: optical coherence tomography angiography; PDR: proliferative diabetic retinopathy. Modified from our unpublished data

DR is the leading cause of blindness among the working-age population and the common microvascular complication in diabetic patients. Based mainly on the microvascular changes (microangiopathy) caused by hyperglycemia, DR is divided into two stages, i.e., non-proliferative DR (NPDR) and proliferative DR (PDR). The pathological lesions in NPDR, such as microaneurysms, vascular leakage, hemorrhages and hard exudates, venous beads, and intraretinal microvascular abnormalities (IRMA), are mainly caused by the altered blood flow and increased vascular permeability in retina [1], the increased thickness of basement membrane [2, 3], loss or dropout of endothelial cells and pericytes [4, 5], and acellular capillary formation [6], which are induced by the chronic hyperglycemia. With the increasing retinal ischemia and disease progression, DR may develop from NPDR to PDR (Fig. 1), which presents a substantial risk for visual loss due to neovascularization on the optic disc or in retina, vitreous hemorrhages, or retinal detachment caused by the retinal fibrovascular proliferation and traction [7]. DR has long been recognized as the microangiopathy. With the recent rapid advancements in the basic research and clinic, DR is also characterized as retinal neurodegeneration and low-to-moderate inflammation, involving all kinds of retinal cells, multiple factors and pathways, which makes the pathogenesis of this disease more complex. Nowadays, DR has been considered as the disease or dysfunction of neurovascular unit (NVU) in retina as a result of chronic hyperglycemia [8].

With the increasing severity of DR, diabetic macular edema (DME) appears more frequently, resulting the visual impairment in patients with DR. In normal retina, the maintenance of fluid entry and exit is controlled by the blood-retinal barrier (BRB) and the normal functions of Müller glias and retinal pigment epithelium (RPE). In the pathogenesis of DME, the increased vascular leakage into the retinal parenchyma and the decreased drainage function of Müller glias and RPE cells results in the imbalance of the retinal fluid influx and efflux, leading to fluid accumulation in macular region. According to the Starling equation, the fluid accumulates intraretinally or subretinally, resulting in retinal thickening and DME formation (Fig. 1). At the molecular level, vascular endothelial growth factor (VEGF) and other cytokines are implicated and involved in the pathogenesis of DME, and the baseline levels of the cytokines in aqueous could be served as the predictors for the response of anti-VEGF treatment in DME [9].

**Fig. 2:**
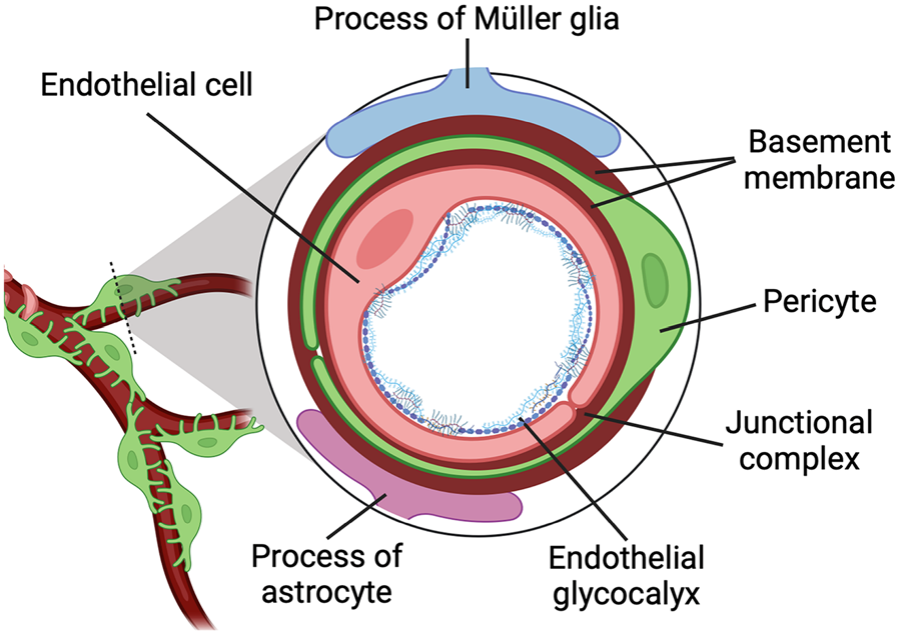
The vascular unit as the basic unit for iBRB to maintain microvascular stability in retina. The vascular unit consists of both cellular (endothelial cells, pericytes, Müller glias and astrocytes) and noncellular (endothelial glycolycax and basement membrane) components, which together maintain the integrity of iBRB. In retinal vascular unit, endothelial cells and pericytes are the two main cellular constituents. The endothelial cells comprise the innermost lining that contacts directly to the blood, forming the first cellular barrier. The endothelial cells form the junctional complexes, composing of tight junctions, adherens junctions and gap junctions. These junctional complexes are the molecular basis for the iBRB integrity, establishing a physical barrier to solutes and water. Pericytes, sharing the basement membrane with endothelial cells, are in close contact with the endothelial cells (pericyte-endothelial interactions). Müller glias physically ensheath blood vessels with their processes, forming Müller glia-vascular cell interactions. Astrocytes are closely associated with the superficial capillary plexus in retina, forming astrocyte-vascular cell interactions. The extracellular matrix surrounding the endothelial cells and pericytes are of importance to maintain the structure and function of iBRB. The endothelial glycocalyx is a dynamic jelly-like and hair-like protective layer, functioning as a shield and barrier, which prevents the adhesion of leukocytes and platelets. The endothelial glycocalyx is composed of a polysaccharide structure, consisting of soluble proteins, glycolipids, glycoproteins and proteoglycans, which forms a polymeric sugar-rich network covering the luminal surface of the endothelial cells. The loss of endothelial glycocalyx contributes to endothelial dysfunction in DR. The vascular basement membrane serves as the substratum to attach the endothelial cells on the luminal surface and pericytes on the abluminal surface of capillary endothelial cells. The vascular basement membrane is mainly composed of collagen IV, laminin, fibronectin, and perlecan, assembling in a highly organized manner. The vascular basement membrane is a ubiquitous, multicomponent, ultrastructural layer that functions as a barrier of selective permeability and mediates the interactions for the matrix and cells. In DR, the basement membrane is thickened, which may alter the substances exchange between the capillaries and retinal parenchyma, and affect the vascular cell viability. DR: diabetic retinopathy; iBRB: inner blood-retinal barrier. This figure was created with BioRender.com

In normal retina, both the cellular (endothelial cells, pericytes, Müller glias, and astrocytes) and noncellular (the endothelial glycocalyx and basement membrane) components form the basic vascular unit (Fig. 2). Within this intact vascular unit, the intercellular communications, the multiple molecule and extracellular matrix (ECM) protein interactions and the microvascular stability are well maintained. DR can affect multiple cellular and noncellular components through different mechanisms, leading to the microvascular destabilization. Microvascular destabilization is the primary cause for inner BRB (iBRB) breakdown, vascular leakage, and the dysfunction of inner retinal NVU (iNVU) in DR and DME. In this review, we discussed the cellular and noncellular components of the retinal microvasculature, the altered extracellular matrix (endothelial glycocalyx and vascular basement membrane) and cell–cell contacts, as well as the changed cytokines and signaling pathways, which cause the microvascular destabilization, leading to the breakdown of iBRB and the dysfunction of iNVU in DR and DME.

## Search strategy and selection criteria

A computer-based online search of the database from PubMed/MEDLINE was used to retrieve articles exploring vascular destabilization in DR with the aim to identify English language articles that were published up to June 6, 2024. The combinations of the following words (MeSH terms) were used to maximize the specificity and sensitivity, including “diabetic retinopathy”; “diabetic macular edema”; “blood-retinal barrier”; “neurovascular unit”; “vascular destabilization”; “endothelial cell”; “pericyte”; “Müller glia”; “astrocyte”; “microglia”; “cell–cell contact”; “vascular endothelial growth factor”; “placental growth factor”; “angiopoietin”; “Tie2”; “vascular endothelial protein tyrosine phosphatase”; “platelet-derived growth factor”; “transforming growth factor-β”; “protein kinase C”; “semaphorin 4D”; and “ephrin-B2”. The papers were further screened by the title and abstract, and those related to vascular destabilization in DR were included to summarize the contributions to vascular destabilization in DR. The papers or articles related to letters, case reports, and the Ph.D. theses were excluded.

## The iBRB maintains the homeostasis and the iNVU function in retina

### The iBRB consists of both cellular and molecular components

**Fig. 3:**
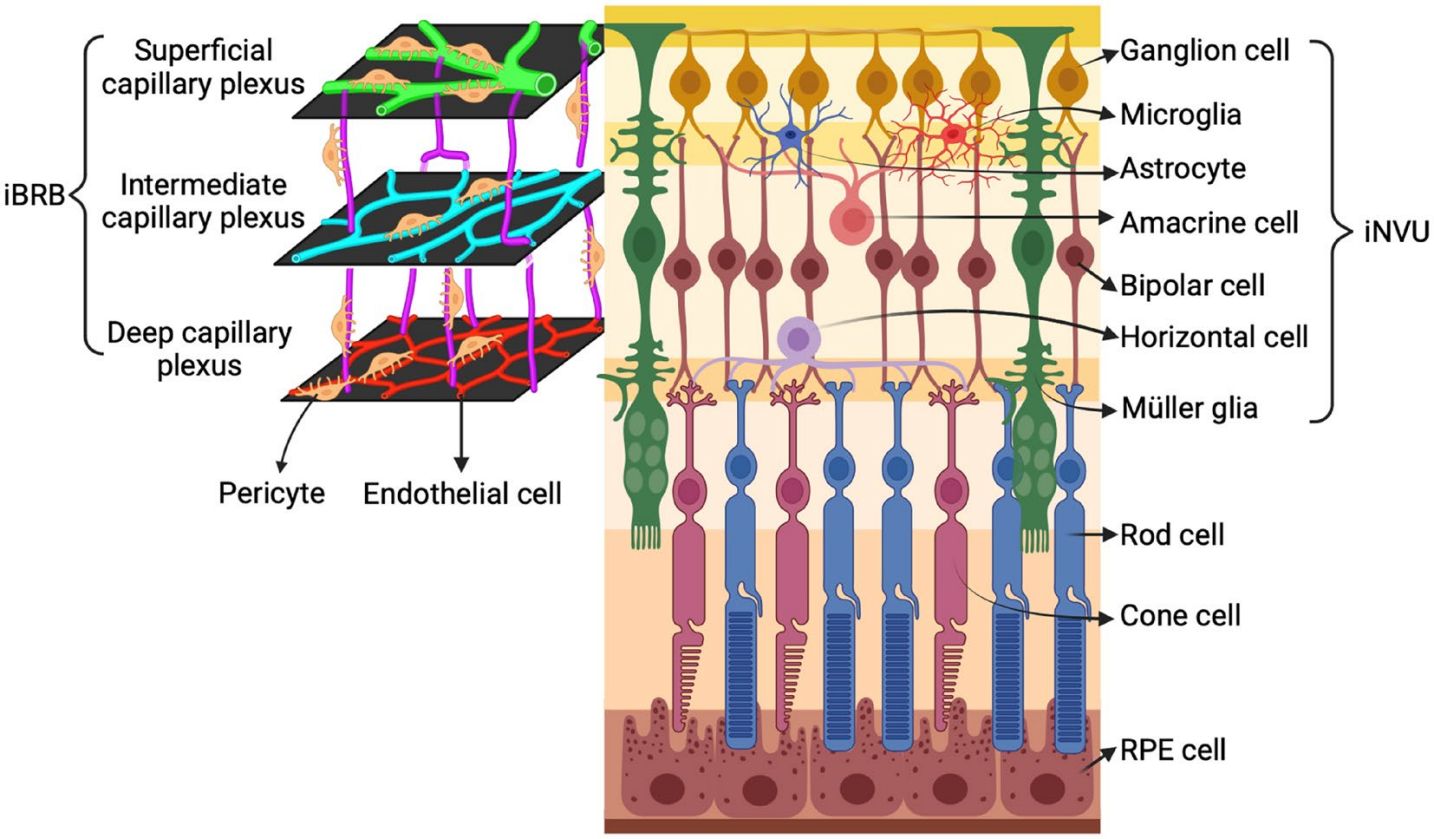
The retina structure and the cellular components of iBRB and iNVU. The retina consists of four types of cells, which assemble in a highly organized manner. These retinal cells are as follows, i.e., (1) the retinal vascular cells (endothelial cells and pericytes), (2) retinal neurons (retinal ganglion cells, amacrine cells, horizontal cells, bipolar cells and photoreceptors), (3) glial cells (Müller glias, astrocytes and microglias), and (4) the retinal pigment epithelial cells (RPE cells). The retina is nourished by two systems, i.e., the central retinal artery suppling the inner retina and the choriocapillaris from the choroidal vasculatures suppling the outer retina and RPE. Both iBRB and iNVU are as the functional unit in retina, in which the integrity of iBRB is of great importance to maintain the normal function of iNVU. The cellular components of iBRB consist of endothelial cells, pericytes, Müller glias and astrocytes. The retinal blood vessels form three layers of vascular beds within the retina, i.e., the superficial capillary plexus, intermediate capillary plexus, and deep capillary plexus, together composing iBRB to maintain the retinal homeostasis and the proper function of the iNVU. The components of iNVU comprise the retinal vascular cells (endothelial cell and pericyte), retinal neurons (retinal ganglion cells, amacrine cells, horizontal cells, and bipolar cells), glial cells (Müller glias, astrocytes and microglias), as well as the ECM proteins. ECM: extracellular matrix; iBRB: inner blood-retinal barrier; iNVU: inner neurovascular unit; RPE: retinal pigment epithelium. This figure was created with BioRender.com

In normal retina, retinal microvessels form the intact iBRB to maintain the normal function of the retina, which provides crucial support for the formation, maintenance, and stability of iNVU. Different to choriocapillaris, the retinal endothelial cells are not-fenestrated, which prevent the mass leakage into the retinal parenchyma from the retinal blood vascular system. The retinal vascular endothelial cells are covered by pericytes and surrounded by glial cell processes. The retinal endothelial cells are coated and ensheathed by pericytes (about 95% coverage) [10]. Retinal endothelial cells and pericytes, together with the surrounding Müller glias and astrocytes, form the cellular components of iBRB (Fig. 3). The pericytes surrounding the endothelial cells are further ensheathed by the processes of Müller glia and astrocytes, which form a continuous layer outside of the retinal blood vessels. The normal function and intact structure of the neuro-glio-vascular cross-talk contributes to the normal function of the iBRB and iNVU. Any disruption to the cellular components of iBRB will finally cause microvascular destabilization in retina.

**Fig. 4:**
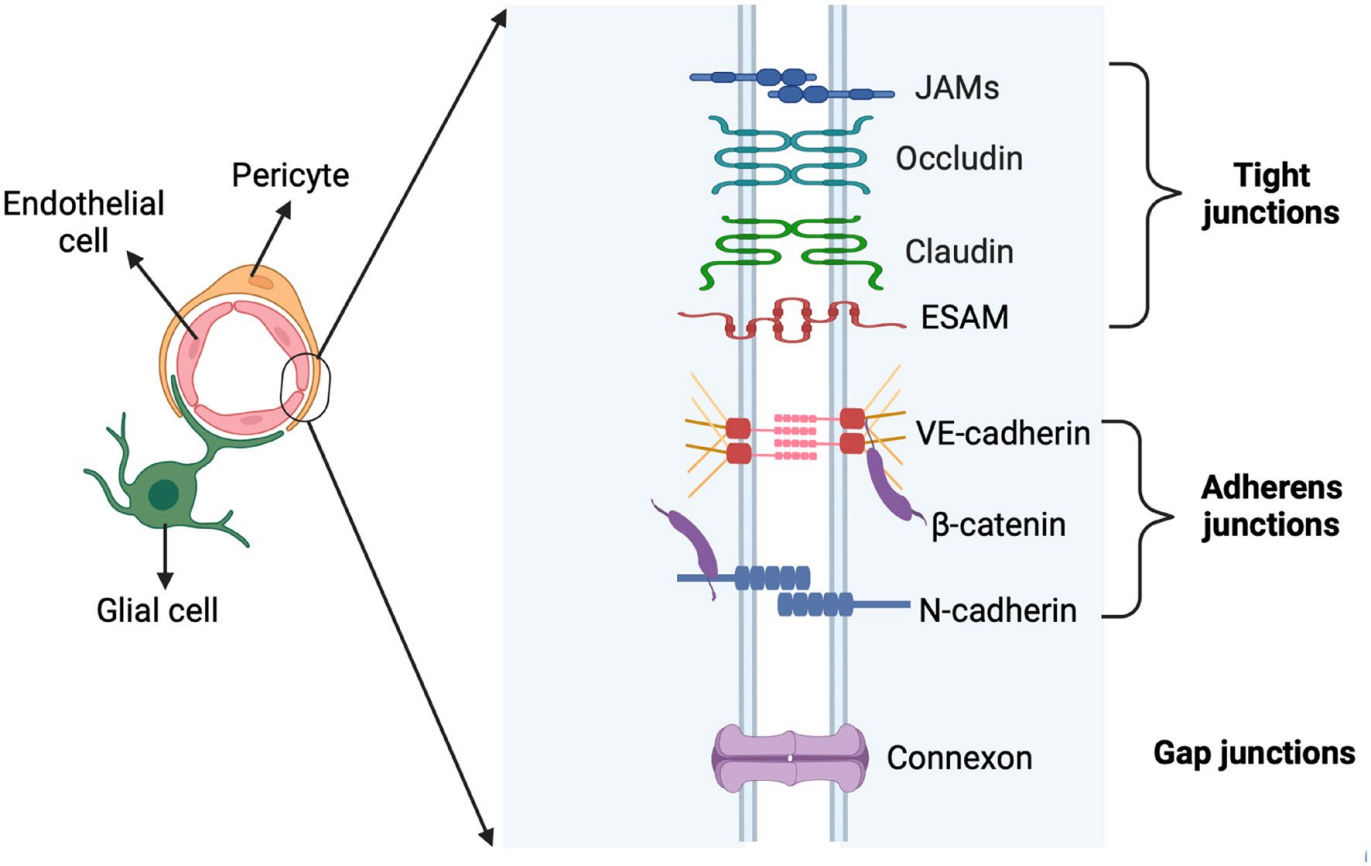
The intercellular junctional complexes between the endothelial cells in the retinal vascular unit. The intercellular junctional complexes are formed between the retinal endothelial cells, which comprise the tight junctions, adherens junctions and gap junctions. Tight junctions are composed of occludin, claudins, JAMs, and ESAM. Adherens junctions consists of VE-cadherin, N-cadherin and β-catenin. Gap junctions are formed by connexon, which is assembled by 6 connexins. The junctional complexes between the retinal endothelial cells are the molecular basis for the integrity of iBRB, which establish a physical barrier to solutes and water. ESAM: endothelial cell-specific adhesion molecule; iBRB: inner blood-retinal barrier; JAMs: junctional adhesion molecules; N-cadherin: neural-cadherin; VE-cadherin: vascular endothelial-cadherin. This figure was created with BioRender.com

The iBRB is ensured by the junctional complexes (Fig. 4), including both tight junctions and adherens junctions, between the retinal endothelial cells on their luminal side, which lay down the molecular basis for the integrity of the iBRB [11–13]. The tight junctions are the transmembrane proteins, consisting of claudins, occludin, and the junctional adhesion molecule (JAM) family proteins [14]. The tight junctions associate with the adaptor proteins, including zonula occludens-1, -2, and -3 (ZO-1, -2, and -3) and cingulin [15, 16]. These intercellular junctional complexes are dynamically regulated by pericytes, astrocytes and Müller glias [12, 17, 18]. Dysfunction or breakdown of the iBBB is mainly due to the reduction of the junctional complexes, which leads to the increase of retinal vascular leakage and the transcellular transport across the endothelium, as well as the infiltration of inflammatory cells into the retina.

### The cellular components of iNVU

The integrity of iBRB maintains the proper function of the iNVU. The iNVU (Fig. 3) comprises retinal vascular cells (endothelial cell and pericyte), retinal neurons (ganglion cell, amacrine cell, horizontal cell, and bipolar cell), glial cells (Müller glia, astrocyte and microglia), as well as the ECM proteins [8]. Retinal vasculatures, consisting of arterioles, capillaries, and venules, are formed by the tightly connected endothelium, lining the luminal side of the blood vessels and facing the blood directly, which forms the first cellular barrier. The endothelium is closely ensheathed and wrapped by pericytes and the foot processes of glial cells (astrocyte and Müller glia), which nourish the retinal neurons. The superficial capillary plexus is surrounded by both Müller glias and astrocytes, but the deep capillary plexus is only ensheathed by Müller glias [19].

In the neurosensory retina, there are three types of glial cells with different locations and functions (Fig. 3). Astrocytes distribute in the inner retina with their processes surrounding the superficial capillaries. Müller glias span the entire retina from internal limiting membrane to the external limiting membrane, with localizing their cellular bodies in the inner nuclear layer. Müller glias contacts with all the neurons and blood vessels in the retina. Microglias are located mainly in the inner retina with ramified morphology in resting state. Microglias are active sensors of the iNVU microenvironment, playing as the tissue macrophages with their long process continuous screening the retina. Microglias become activated when responding to the insults, which transforms into the reactive phagocytes with the ameboid morphology [20].

In DR, iNVU dysfunction is manifested as iBBB breakdown, neurovascular dysregulation, retinal cell death, reactive gliosis, and microglial activation. The proper function of iNVU can maintain and coordinate the neurovascular coupling in retina.

### The functional hyperemia as the atuoregulation of neurovascular coupling in retina

Retinal blood vessels lack autonomic innervation [21], which is different to the choroidal vasculatures. However, there exists autoregulation in the retinal circulation [22]. As one of the example of the autoregulation of neurovascular coupling in retina, the functional hyperemia, i.e., increase in blood flow, is well developed in the retinal vasculature by the light stimulation [23]. The response of functional hyperemia can supply both glucose and oxygen to the retinal neurons to meet their metabolic needs [24]. The decreased functional hyperemia was reported as the early change in DR, including the changes of basal blood flow [19, 24], which indicates the decreased neurovascular coupling in iNVU. The detailed mechanisms for the reduced functional hyperemia still remain largely unknown [19].

### The iBRB breakdown leads to microvascular destabilization and iNVU dysfunction in DR

DR is mainly characterized by microvascular alterations, including basement membrane thickening, disruption or loss of tight junctions between endothelial cells, loss or dropout of pericytes and endothelial cells, acellular capillary formation, increased vascular permeability, capillary occlusions, microaneurysms, IRMA, and etc. Diabetes impacts the iBRB, which causes the dysregulation of neurovascular coupling and results in iNVU dysfunction. In DR, the altered iBRB function as the biomarker of microvascular destabilization is characterized as the increase in vascular permeability and vascular abnormalities, and the dysregulation of retinal blood flow. The decreased microvascular stability, culminating in vascular cell loss, increased permeability, inflammation, and even neovascularization in response to hyperglycemia and hypoxia, leads to decreased ability of the retinal blood vessels to meet the needs of the neurosensory metabolism, further aggregating the function of iNVU.

Therefore, the breakdown of iBRB and dysfunction of iNVU can cause both microangiopathy and neurodegeneration in DR, which might be considered as therapeutic targets. As the basic unit of iBRB and iNVU, the vascular unit (Fig. 2) contains extracellular matrix, including endothelial glycocalyx and vascular basement membrane, to maintain the homeostasis and normal function of iBRB and iNVU.

## The extracellular matrix of the retinal vascular unit in normal and diabetic retina

The endothelial cells and pericytes are sandwiched between endothelial glycocalyx and basement membrane of the blood vessels. They also share the same basement membrane between them. The extracellular matrix surrounding the endothelial cells and pericytes are of importance to maintain the structure and function of iBRB. Any disturbance to the extracellular matrix will result in the endothelial dysfunction and leads to iBRB breakdown in DR.

### Endothelial glycocalyx as a shield and barrier for endothelial cells

The endothelial glycocalyx, a dynamic jelly-like and hair-like protective layer, is a polymeric sugar-rich network covering the luminal surface (apical side) of the endothelium (Fig. 2). The endothelial glycocalyx interacting with several plasma factors forms a physiological layer [25], and works as a shield separating the endothelial cells from the circulating blood. The endothelial glycocalyx has many functions, including regulation of the vascular permeability and inflammation and facilitation of the interactions of plasma factors with the receptors in endothelium.

#### The composition of endothelial glycocalyx

The endothelial glycocalyx is composed of a polysaccharide structure, consisting of soluble proteins, glycolipids, glycoproteins and proteoglycans [26, 27]. The proteoglycans and glycoproteins are the main molecules, serving as “backbone” to mediate the glycocalyx binding to the membrane endothelial cells. Proteoglycans have the glycosaminoglycan (GAG) side-chains, which are long and unbranched; while glycoproteins have carbohydrate side-chains, which are short and branched [28]. Proteoglycans is composed of the core proteins, including transmembrane (syndecan 1–4) and glycosylphosphatidylinositol anchored (glypican 1–6) proteins, which bind to GAGs covalently [28]. The most abundant GAGs include chondroitin sulfate, heparan sulfate, and hyaluronic acid [29]. Besides, hyaluronic acid is bound to cluster of differentiation (CD) 44 on the endothelial membrane and provides the structural support for the glycocalyx [30].

The plasma proteins, such as albumin, anti-thrombin III, sphingosine-1-phosphate (S1P), and superoxide dismutase, are found to be integrated into the network of glycocalyx with different functions. The albumin can reduce the hydraulic conductivity and prevent the degradation of the glycocalyx [31, 32]. S1P, synthesized by red blood cells and delivered by plasma albumin and high-density lipoprotein (HDL), can stabilize the glycocalyx cytoskeleton, and regulate the glycocalyx composition and the intercellular junctions.

The components of endothelial glycocalyx changes dynamically. Besides the synthesis and organization, the components within glycocalyx are cleaved and shed from the endothelial surface by different enzymes, e.g., heparinase cleaving heparan sulfate, hyaluronidase cleaving hyaluronic acid, and matrix metalloproteinases (MMPs) cleaving proteoglycans and glycoproteins.

#### The function of endothelial glycocalyx

The endothelial glycocalyx functions as a shield and barrier, preventing the adhesion of leukocytes and platelets; acts as mechano-transduction of shear forces, stimulating nitric oxide (NO) production upon the shear stress; and also regulates red blood cell distribution and tissue oxygenation [27, 33–35]. The glycocalyx confers semi-permeability due to the characteristics of the electrical charge. The abundant sulphate residues on GAG side-chains and the carboxyl groups in hyaluronic acid accounts for the net negative charges on the endothelial glycocalyx, which favors the binding of the positively charged molecules [36]. The negative charge of hyaluronic acid can also provide the endothelial glycocalyx with exceptional hydration properties. The endothelial glycocalyx, together with the endothelial junctional complexes, plays the important role to regulate vascular permeability [37, 38].

#### The loss of endothelial glycocalyx contributes to endothelial dysfunction in DR

In DR, the glycocalyx loss in retinal vascular unit was detected both experimentally and clinically, which could attenuate the functions of the endothelial glycocalyx substantially. The glycocalyx loss will increase vascular leakage and retinal edema, impair the normal vascular response to shear stress, and enhance leukocyte adhesion to the endothelial cells [39]. For example, it was reported that the vascular leakage was increase by 3.6-fold after the endothelial glycocalyx removal [40].

In diabetic patients, oral sulodexide (200 mg/day, a commercially available compound consisting of heparan sulphate and dermatan sulphate) for two months, can increase retinal glycocalyx dimensions in diabetic patients, maintain the vascular integrity as well as decrease the plasma hyaluronidase [41]. However, sulodexide cannot provide sufficient efficacy to reduce the renal complication caused by diabetes [42]. Several therapeutic strategies targeting the endothelial glycocalyx are reviewed [33, 35, 43], which deserved the clinical study to test their effect on DR as well as other vascular diseases.

#### The mechanisms for endothelial glycocalyx loss in DR

The degraded glycocalyx or decreased glycocalyx density has been observed in experimental DR and diabetic patients. High glucose can decrease the synthesis and sulfation of proteoglycans, and reduce GAG incorporation into core proteins, thus leading to the reduction of the endothelial glycocalyx synthesis. Furthermore, high glucose can increase the levels of sheddases (heparanase, hyaluronidase and MMPs), which are responsible for the degradation and decreased density of endothelial glycocalyx [39]. Thus, reduced synthesis and increased shedding can result in the loss of glycocalyx, leading to the microvascular destabilization [39].

The elevated levels of heparanase and subsequent increased glycocalyx and occludin shedding were reported by using high glucose-treated human retinal endothelial cells [44]. In vitro study using high glucose treated rat retinal microvascular endothelial cells, the shedding of glycocalyx components, including heparin sulfate, chondroitin sulfate, and hyaluronan, were detected to be increased [45]. In diabetic rat and mouse models, the endothelial glycocalyx size was significantly decreased in retinal vessels [46]. A reduction of endothelial glycocalyx volume, accompanied with the increased hyaluronan catabolism, i.e., elevation of plasma hyaluronan and hyaluronidase, was also evidenced in the retinal blood vessels from patients with type 2 diabetes [41]. The increased levels and activities of sheddases including heparanase [47], hyaluronidase [35, 40], and MMPs [48], were reported in the plasma and vitreous of the diabetic patient. Besides, hyperglycemia increased leukocyte adhesion by increasing intercellular adhesion molecule 1 (ICAM-1) and producing the MMPs (MMP-2 and -9), which also cause shedding and cleavage of the glycocalyx, leading to endothelial dysfunction [49, 50].

### Vascular basement membrane as a physical barrier for retinal blood vessels

The vascular basement membrane is a multifunctional unit, providing the structural integrity to microvasculature and displaying various functions. Basement membrane serves as the substratum to attach the endothelial cells on the luminal surface and pericytes on the abluminal surface of capillary endothelial cells (Fig. 2).

#### The components of vascular basement membrane

The main components of vascular basement membrane include collagen IV, laminin, fibronectin, and perlecan, which assemble in a highly organized manner [3]. The assembly of basement membrane is well-regulated, involving the degradation by MMPs as well as the synthesis and deposition of the new components [3]. Assembly of the basement membrane is achieved via the interactions between different basement membrane components and their interactions with molecules on the cell membrane [51]. Both endothelial cells and pericytes contribute to the synthesis of basement membrane components, and loss of these cells can lead to capillary hyperdilation, impacting retinal blood flow negatively in DR [52].

#### The function of vascular basement membrane

The vascular basement membrane is a ubiquitous, multicomponent, ultrastructural layer that functions as a barrier of selective permeability and mediates the interactions for the matrix and cells. The basement membrane is involved in many functions to maintain the microvascular stability, regulating cell death, the contractility of pericytes, vascular permeability, neovascularization, and the cell–cell communications [3]. In brain, the vascular basement membrane acts as a passage transporting fluid through the blood vessels [53], while the perivascular basement membrane and the ECM proteins support the interface between the glial cells and vascular cells [54]. The functions of these basement membrane in brain are much similar to those in retina. In retina, the tight junctions between the endothelial cells ensures iBRB and controls vascular permeability. ECM proteins, such as collagen IV and laminin, contribute to the proper organization of tight junctions, indicating the contribution of the basement membrane to preserve the selective permeability of iBRB [55].

#### The effect of basement membrane thickening in DR

The thickening of basement membrane contributes to the microvascular pathologies in diabetes, which could be evidenced in most tissues [3], such as in the retina [56], kidneys [57], muscle [58], and skin [59]. Thickening of vascular basement membrane is well studied and documented both in diabetic animal models and diabetic patients [3]. The basement membrane, examined with electron microscopy, was much more thickened in the retinal capillaries of DR patients when compared with the non-diabetic subjects, showing that the thickened basement membrane in retinal capillaries is a characteristic feature in DR [60]. One study showed that the thickness of capillary basement membrane was increased significantly in diabetic patients compared with controls (583.1 nm ± 38.52 *vs* 292.4 nm ± 24.3), with more significant thickening in external basement membrane of the blood vessels [56].

The thickened basement membrane may alter the substances exchange between the capillaries and retinal parenchyma, and affect the vascular cell viability. Basement membrane thickening increased the vascular permeability and leakage, and also promoted cell apoptosis [3]. Accumulating data showed that basement membrane thickening can impact the cellular communications, modulating the cell metabolism and substances exchange, thereby impact the cell survival and neovascularization in DR [3]. In addition, high glucose can stiffen the basement membrane and alter the vascular elasticity, thus compromising the regulatory ability of the pericytes to the retinal blood flow [61]. This can further promote the activation endothelial cells, which mediate the inflammation by increasing ICAM-1 and promoting leukocyte adhesion in retina [61].

#### The mechanisms for the basement membrane thickening in DR

Basement membrane thickening is due to the increased synthesis as well as the decreased degradation of the basement membrane components under diabetic conditions [3]. The increased polyol pathway can lead to the polyalcohol accumulations in vascular cells and alter the enzymes activities in the synthesis of basement membrane [62, 63]. The activity of the enzymes involved in basement membrane catabolism was reduced [62]. The non-enzymatic glycosylation and oxidative modification also contribute to the susceptibility to proteolytic resorption of basement membrane in diabetic patients [64]. Besides, connective tissue growth factor (CTGF) promotes the thickening of capillary basal lamina and pericyte loss, destabilizing the blood vessels in DR [65]. One study using transmission electron microscopy observing the enucleated human eyes showed that excessive basement membrane materials were deposited in retinal capillaries by Müller cells during aging and diabetes [56].

Both endothelial glycocalyx and vascular basement membrane provides structural supportive roles for the homeostasis and normal function of the vascular unit, which maintains the microvascular stability. Besides the extracellular matrix, the cell–cell contracts are also of great importance to maintain the microvascular stability of the retinal vascular unit.

## The cell–cell contacts are altered in DR

As described above, many retinal cells constitute iBRB and iNVU. The interactions between these cells play the pivotal roles for the normal functionality of iBRB and iNVU. The complex interactions within iNVU requires each cell operating in a well-coordinated manner to ensure the control of retinal homeostasis by the iBBB (Fig. 3). The complex interactions include direct (i.e., endothelial-to-endothelial cell, glial-to-glial cell, pericyte-to-endothelial cell, and cell–matrix interaction) and indirect (i.e., Müller glia-to-endothelial cell, microglia-to-endothelial cell) interactions [66], which are discussed below.

### Endothelial-endothelial contact serves as the basis for iBRB integrity

Endothelial cells comprise the innermost lining that contacts directly to the blood, forming the first cellular barrier. The endothelial-endothelial contact serves as the basis for iBRB integrity, which restricts the free movement of the most molecules including proteins, lipids, glucose and solutes into or out of the retinal parenchyma. The endothelial cells act as a permeable filter, allowing the selective substances exchange between the luminal and abluminal sides of the endothelium. Loss of highly regulated endothelial-endothelial contacts results in the vascular dysfunction and ultimately the iBRB breakdown, causing vascular hyperpermeability and macular edema in DR. Multiple factors including but not limited to cytokines and growth factors [9], inflammation-related factors [67, 68], chemokines, altered expression of receptors and transporters [69], advanced glycosylation end products and receptors, and oxidative stress, and etc., could promote retinal endothelial dysfunction in DR [49], resulting in microvascular destabilization and aggravating the disease progression. In a recent study, porous Se@SiO2 nanospheres were demonstrated to protect the retina and retinal endothelial cells against diabetic insults both in vivo and in vitro through suppression of lipid peroxidation and inflammation by targeting glutathione peroxidase 4 (GPX4), showing the promising potency for the treatment of DR [70].

#### Junctional complexes ensure iBRB integrity

Endothelial junctions are sealed by the junctional complexes (Figs. 2 and 4), composing of tight junctions, adherens junctions and gap junctions [71–73]. These junctional complexes (Fig. 4) are the molecular basis for the iBRB integrity, establishing a physical barrier to solutes and water [11–13, 18]. In normal retina, the endothelial cells lack fenestrations, only having few pinocytotic vesicles. The intact endothelial cells together with the elaborate tight junctions to maintain the iBRB integrity [12].

As shown in Fig. 4, the junctional complexes are depicted. Tight junctions are composed of occludin, claudins, junctional adhesion molecule (JAM)-A (JAM-A), JAM-C, and endothelial cell-specific adhesion molecule (ESAM) [18]. Adherens junctions consists of vascular endothelial-cadherin (VE-cadherin), neural-cadherin (N-cadherin) and β-catenin [12, 18]. Gap junctions are formed by connexon, which is assembled by 6 connexins (cx). Gap junctions facilitate the electrical and chemical communications between cells and permit the free passage of small molecules (< 1 kDa). Among the connexins, connexin-7, -40, and -43 (cx-7, cx-40 and cx-43) are mainly expressed in the retinal endothelial cells [74, 75].

The changes of junctional proteins via decreased protein expression and increased the phosphorylation are the major contributing factors to increase vascular permeability in several vascular disorders [11, 76, 77].

#### Two transport routes of endothelial cells in normal retina

Endothelial cells use two transport routes to control the molecule passage from the blood into the retinal parenchyma, i.e., the paracellular route and the transcellular route. As mentioned above the paracellular route is ensured by tight junctions and enhanced by adherens junctions. The transcellular transport is mainly dependent on the selective transport by caveolar vesicles, especially caveolin-1 (Cav-1) [78]. Besides, the retinal endothelial cells express less vesicle transporters and more efflux pumps, together regulating the transcellular transport across the endothelium in normal retina [16].

#### Increased transport across the endothelial cells in DR

Hyperglycemia-induced vascular permeability can result from the increase of both paracellular and transcellular transports across the retinal endothelium, through the opening or disruption of endothelial intercellular junctional complexes and/or the increased caveolar transcellular transport in endothelial cells [12, 18], thus resulting in the consequence of retinal leakage and edema. For example, hyperglycemia down-regulates the expressions of claudin-5, occludin, JAM-A, and ZO-1 in human retinal endothelial cells [79, 80] and decreases the VE-cadherin phosphorylation via the increasing Ang-2 [81]. In vivo study demonstrated that VEGF-induced vascular permeability in non-human primates was due to the increased transcellular transport, which was through the endothelial NO regulated caveolae, but not the opening of tight-junctions or the increased fenestrations [82, 83]. The plasmalemma vesicle-associated protein (PLVAP) is involved in VEGF-induced iBRB breakdown in DR [84]. In Cav-1 knockout mice, Cav-1 inhibition induces iBRB breakdown without alterations of tight junction protein expressions, indicating that alteration of vesicular transport could enhance the vascular permeability in the retina [78]. Other factors, such as tumor necrosis factor α (TNFα), placental growth factor (PlGF), transforming growth factor-β (TGF-β), MMP-9, VEGF, could decrease or disrupt the tight junctions via direct and indirect mechanisms in DR [18, 85].

#### Multi-modes of retinal endothelial cells death aggravate iBRB breakdown

Endothelial dysfunction is an important pathological feature of DR, to which endothelial cell death contributes more. Loss of endothelial cells (cell death) further disrupted iBRB, causing iBRB breakdown and increasing the acellular capillaries formation.

Endothelial cell death was observed in retina of diabetic patients and diabetic rat model [86]. Growing evidence shows that multi-modes of cell death, such as apoptosis [85, 87], necroptosis [88], ferroptosis [89, 90], pyroptosis [91, 92], and parthanatos [93], are associated with retinal endothelial cell death in DR [4, 94]. Oxidative stress, leukostasis, and inflammation are the main underlying mechanisms for retinal endothelial cell death. In streptozotocin-induced diabetic rats, iBRB breakdown was caused by the endothelial cell apoptosis, which was through leukocyte-mediated Fas-FasL-dependent pathway [95]. Under diabetic conditions, TGF-β released by the activated macrophages increases the pro-apoptotic protein TGF-β-induced Gene Human Clone 3 (BIGH3) in retinal endothelial cell, which further induces retinal endothelial cell apoptosis through an autocrine loop [96]. Besides, we previously reported that the microglia become activated in experimental DR, and it can phagocytose the endothelial cells through penetration of the retinal vascular basement membrane, leading to iBRB breakdown and acellular capillaries formation [97].

### Pericyte-endothelial interaction maintains the microvascular stabilization

In retinal microvasculature, pericytes and endothelial cells are the two main cellular constituents (Fig. 2). Pericytes, sharing the basement membrane with endothelial cells, are in close contact with the endothelial cells (pericyte-endothelial interactions). Pericytes maintain the microvascular stability, and provide the structural support for the microvasculatures. Direct contact and communications between pericytes and endothelial cells are crucial for the integrity of iBRB and normal function of iNVU [98]. The mature retinal microvasculatures covered with pericytes makes the endothelial cells less responsive to VEGF.

#### Pericyte coverage ensures the microvascular stabilization

Pericytes, the mural cells on capillaries and positioned at the abluminal surface of endothelial cells, enwrap the retinal microvessels along with endothelial cells, which separate endothelial cells from other cells within the iNVU. Pericytes play a pivotal role in microvascular stabilization, regulating the retinal capillary blood flow and the proliferation of endothelial cells [99].

**Fig. 5:**
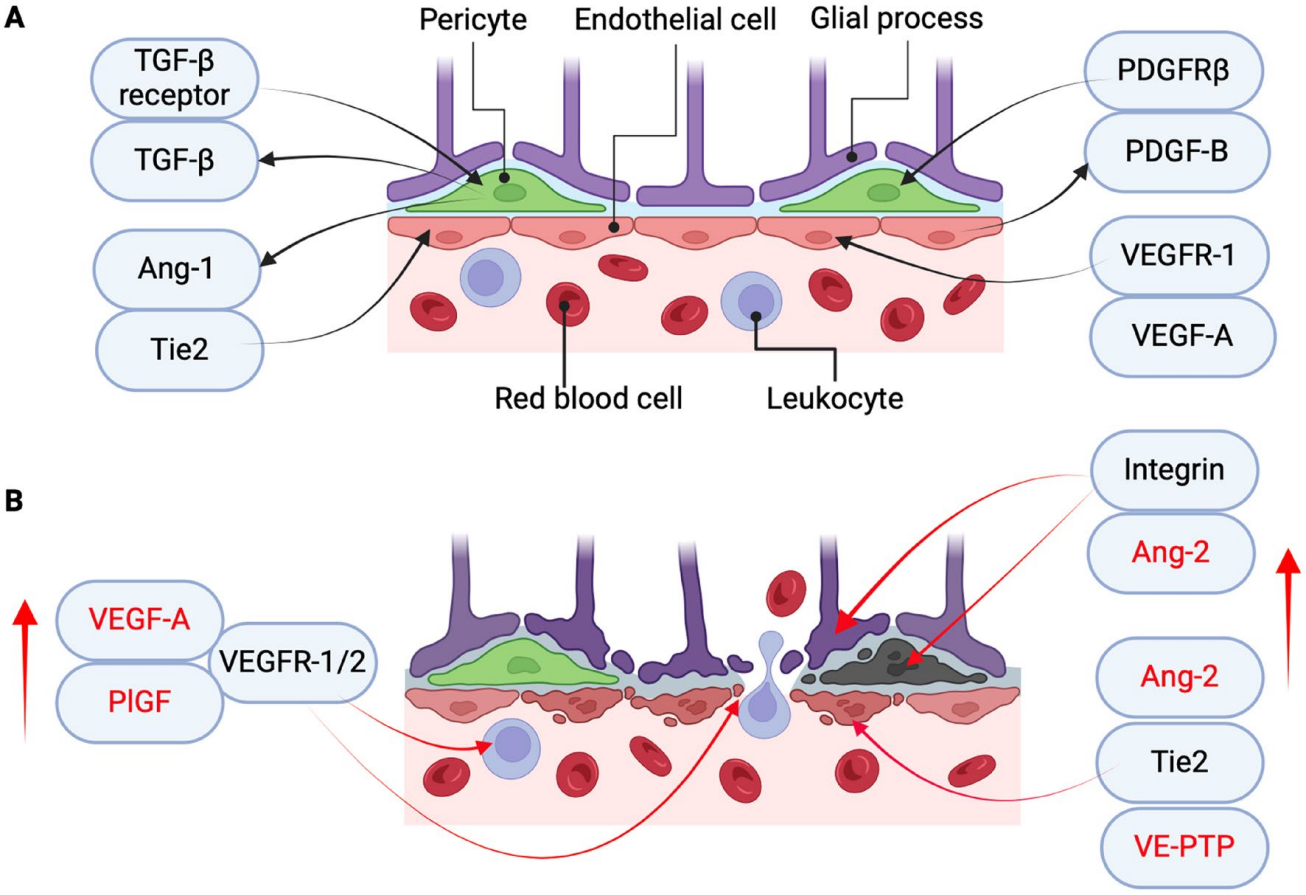
The molecular interplays between pericytes and endothelial cells. **A** In normal retina, the survival of endothelial cells is supported by VEGF-A/VEGFR-1. The microvascular stabilization is maintained together by endothelial cells-derived PDGF-B, which recruits the coverage of the pericytes through binding to PDGFRβ on pericytes. The pericytes secreted Ang-1, promoting the vascular stabilization via binding Tie2 on the endothelial cells, and TGF-β, promoting pericyte differentiation through binding to TGF-β receptors on pericytes. **B** In DR, hypoxia and hyperglycemia increased the expressions of various cytokines and growth factors, such as VEGF, PlGF, Ang-2, and VE-PTP. VEGF and PlGF can activate the endothelial cells, break down the iBRB, and promote the inflammation through binding to VEGFR-1/2 on the endothelial cells and the inflammatory cells. PlGF induces the pericyte dropout via binding to VEGFR-1 on pericytes. Ang-2 results in the pericyte and astrocyte loss or dropout through binding to integrin receptors on their cell surface, further sensitizing the endothelial cells to VEGF and PlGF. Ang-2 destabilizes the endothelial cells through binding to Tie2 or integrin, leading to the vascular leakage, inflammation, and even neovascularization. VE-PTP dephosphorylates Tie2 and inactivates Tie2, further causing the microvascular destabilization. Besides, other cytokines, growth factors and inflammatory factors, such as TNFα, IL-1β, IL-6, ICAM-1, VCAM-1, MCP-1, BMP2, BMP4, sSema4D, and S1P, are also involved in the microvascular destabilization. These molecules, working synergistically, form the complex molecular networks and result in the iBRB breakdown, retinal vascular hyperpermeability, inflammation, and neovascularization, aggravating the disease progression of DR. The ligands (e.g., TGF-β, PDGF-B, and Ang-2) and the corresponding receptors (e.g., TGF-β receptor, PDGFRβ, and Tie2) are grouped together in Figure A and B. The arrows from the cells indicate the growth factors or cytokines produced by the cells; while the arrows towards the cells indicate the growth factors or cytokines exert their effects through binding to their corresponding receptors on the target cells. For example, PDGF-B is produced by the endothelial cells, which recruits pericytes through binding to PDGFRβ on pericytes. Ang-1: angiopoietin 1; Ang-2: angiopoietin 2; BMP2: bone morphogenetic protein 2; BMP4: bone morphogenetic protein 4; DR: diabetic retinopathy; ICAM-1: intercellular adhesion molecule-1; IL-1β: interleukin-1β; IL-6: interleukin-6; MCP-1: monocyte chemotactic protein 1; PDGF-B: platelet-derived growth factor; PDGFRβ: platelet-derived growth factor receptor β; PlGF: placental growth factor; S1P: sphingosine-1-phosphate; sSema4D: soluble semaphorin 4D; Tie2: tyrosine kinase with immunoglobulin-like and the epidermal growth factor-like domains 2; TNFα: tumor necrosis factor α; VCAM-1: vascular cellular adhesion molecule-1; VEGF-A: vascular endothelial growth factor A; VEGFR-1/2: vascular endothelial growth factor-1/2; VE-PTP: vascular endothelial protein tyrosine phosphatase. This figure was created with BioRender.com

Close pericyte-endothelial interactions are necessary to maintain the microvascular stability [100]. The essential autocrine and paracrine signaling pathways, such as VEGF, platelet-derived growth factor subunit B (PDGF-B), Notch, angiopoietin (Ang), Norrin, and TGF-β, have been well characterized to regulate their interactions [98, 101]. Similar to that in blood–brain barrier [102], the PDGF-B/PDGF receptor β (PDGFRβ) signaling pathway is pivotal for the proliferation, survival, and recruitment of pericytes during angiogenesis [103]; while angiopoietin-1 (Ang-1)/tyrosine kinase with immunoglobulin-like and the epidermal growth factor (EGF)-like domains 2 (Tie2) signaling pathway is required for vascular stabilization [104]. In an intact iBRB, Ang-1, produced by pericytes, binds to the Tie2 receptor expressed on endothelial cells. Activation of the Tie2 receptor in endothelial cells enhances the expressions of endothelial junctions and also produces PDGF-B, which binds to PDGFRβ on pericytes further strengthening the pericyte-endothelial interactions (Fig. 5).

Circular RNAs (cZNF532 and cPWWP2A) also regulate the pericyte-endothelial interactions. For example, cZNF532, acting as the sponge for miR-29a-3p, increased the expressions of nerve/glial antigen 2 (NG2), lysyl oxidase-like 2 (LOXL2), and cyclin-dependent kinase 2 (CDK2) in pericytes. cZNF532 knockdown or miR-29a-3p overexpression could aggravate pericyte degeneration and vascular dysfunction in streptozotocin-induced DR model [105]. cPWWP2A, acting as the miR-579 sponge, could upregulate the protein expressions of Ang-1, occludin and Sirtuin 1 proteins, and cPWWP2A knockdown or miR-579 overexpression aggravated retinal vascular dysfunction [106].

#### The altered gap junctions in pericyte-endothelial interactions in DR

The pericyte-endothelial interactions are complex, which have been widely explored [75, 98, 101]. The vascular homeostatic maintenance in retina depends on the proper activity of gap junction intercellular communication (GJIC) [75]. Studies have revealed the existence of gap junctions between pericytes and endothelial cells both in vitro and in vivo [107, 108]. The pericyte-endothelial cell junction is formed mainly by N-cadherin and cx-43 [18]. It has been shown that hyperglycemia decreased cx-43 expression or increased cx43 degradation in retinal vascular cells, reducing the GJIC activity and compromising endothelial functions related to iBRB breakdown, and ultimately the death of endothelial cells and pericytes in DR [109–111]. One previous study showed that hyperglycemia decreased cx-43 expression and reduced GJIC activity, which further decreased the expressions of tight junctions, such as occludin and ZO-1, and thereby contributing to the iBRB breakdown [79].

#### Pericyte dropout/death leads to microvascular destabilization

The ratio of pericyte to endothelial cell is crucial for the barrier function in the retina. In normal retina, the ratio of pericyte to endothelial cell is 1:1 [112], covering about 95% of the microvascular area [10], which is essential for vascular stabilization and maturation. In DR, pericytes are highly susceptible to the metabolic challenges, showing selective loss/dropout of pericytes [5, 112], with the ratio becomes 1:4, causing the increased vascular leakage [113].

The reduced pericyte coverage may largely compromise the communications between endothelial cells and pericytes, which will contribute to the microvascular destabilization. In the rat retina, ultrastructural observations showed that the decreased pericyte coverage and the altered pericyte-endothelial relationship during aging and diabetes may cause vascular destabilization [3, 114]. Pericyte coverage and maturation is necessary for vessel remodeling during angiogenesis [115]. Transient inhibition of the pericyte recruitment by the anti-PDGFRβ antibody in rat caused the significant pericyte-endothelial dissociations and the breakdown of BRB, mimicking the characteristic features as seen in DR [116].

Substantial evidence showed that pericyte dropout results in microvascular destabilization, leading to the formation of microaneurysms, hemorrhages, pericyte ghosts, the apoptosis of endothelial cells, and acellular capillaries in DR [117–119]. The microvascular destabilization results in the subsequent retinal ischemia, leading to the increased vascular permeability (macular edema) and angiogenesis, i.e., retinal neovascularization in PDR, and eventually causing severe vision loss [117].

#### The mechanisms causing pericyte dropout/death in DR

Several factors cause pericyte dropout/death in diabetic conditions including hypoxia, advanced glycation end-products (AGEs) accumulation, increased levels of reactive oxygen species (ROS), rapid glycemic variations, macrophages/microglia activation, and activation and increased level of MMPs [18]. AGEs reduce the ratio of Bcl-2/Bax and activate caspase-3 through oxidative stress [120], leading to pericyte cell death. AGEs stimulate pericyte detachment and migration through interaction with CD44 forming the CD44 cluster via increasing moesin phosphorylation through activating Rho-kinase [121].

The angiopoitin-2 (Ang-2)/Tie2, VEGF/VEGF receptor-1 (VEGFR-1) and PDGF-B/PDGFRβ pathways have been studied, which account for pericyte death [122]. Under normal condition, Ang-1/Tie2 pathway is important to maintain the microvascular stabilization [123]. However, under hyperglycemia, endothelial cell-derived Ang-2 causes pericyte death through binding to α3β1 integrin [124]. Circulating VEGF and PlGF could result in pericyte ablation from the retinal vasculature via the VEGFR-1-mediated signaling, increasing the vascular leakage in cancer associated retinopathy [125]. VEGF, through forming the complex with PDGFRβ and VEGFR-2, can negatively regulate pericytes, disrupting its recruitment and maturation [126]. Hyperglycemia increased the expression of Src homology-2 domain-containing phosphatase-1 (SHP-1) through activating protein kinase C (PKC) δ and p38α mitogen-activated protein kinase (MAPK), which induces dephosphorylation of PDGFRβ and results in pericyte apoptosis [127]. Pericyte loss/death was also induced by the activation of Ca^2+^/calmodulin-dependent protein kinase II (CaMKII)-inducible nitric oxide synthase (iNOS) pathway [128], or through increased BIGH3 protein expression in DR [129]. Under diabetic condition, pericytes increased MMP-2 expression further induce its apoptosis/anoikis by loss of appropriate contact with ECM proteins [130].

### Müller glia-vascular cells interactions

Müller glias physically ensheath blood vessels with their processes, forming the iBRB. A close relationship between Müller glias and retinal vascular cells account for the formation and maintenance of the iBRB, nutrients uptake and metabolites disposal in normal retina. Müller glias, spanning the entire neurosensory retina, ensures the intact contact between Müller glias and other retinal cells, including neurons, astrocytes and retinal vascular cells. Müller glias can supply the metabolic substrates to retinal neurons, deactivate and facilitate the neurotransmitters recycling, and ensures the homeostatic maintenance of ion balance in the retina. Müller glias express various ion and aqueous channels, maintaining the homeostasis of the retina, as well as keeping the neural retina relatively dry. Perivascular aquaporin 4, inward rectifier potassium channel 4.1 (Kir4.1), α-syntrophin and the short dystrophin isoform 71 form a macromolecular complex facilitates the fluid transport from the retinal parenchyma to the retinal blood vessels [18, 131, 132].

In vitro study, the barrier function formed by the endothelial cells when co-cultured with Müller glia is decreased under hypoxia [133]. In the streptozotocin-induced diabetic rat, the expression of glial fibrillary acidic protein (GFAP) in Müller glias is altered, accompanied with the decreased expression and redistribution of occludin in retinal endothelial cells [134]. The role of glial cells in the maintenance of iBRB was further confirmed by selective depletion of glial cells. Intravitreal administration of DL-α-aminoadipic acid, ablating astrocytes and Müller glias, increased VEGF expression and decreased claudin-5 expression, causing iBRB breakdown [135]. The specific depletion of Müller glia, using the adeno-associated viral vector to deliver a photo-inducible toxic protein, caused retinal remodeling, abnormal vessels and increased vascular leakage [136].

Gap junction communication between Müller glias and pericytes is essential for their survival. High glucose-induced cx43 downregulation in retinal Müller glias resulted in the impairment of GJIC between co-cultured Müller glias and pericytes, that ultimately contributed to apoptosis of both cells, indicating the cell–cell communication via cx43 is important for the survival of Müller glias and pericytes [111]. CD40 ligation in Müller glia triggers the purinergic receptor-dependent inflammatory responses in microglia/macrophages [137] and also induces retinal endothelial cell death [138], promoting DR progression through CD40-ATP-P2X7 pathway [139].

### Astrocyte-vascular cells interactions

Astrocytes are located in the inner retina, closely associated with the superficial capillary plexus in retina [140, 141] and help to maintain iBRB integrity [142]. Astrocytes maintains the iBRB barrier properties by upregulating ZO-1 expression and modifying endothelial morphology [143]. Dysfunction of astrocytes contribute to iBRB breakdown, resulting in the increased vascular leakage [144, 145]. Diabetes changes the expression of GFAP in retinal glias, which is accompanied by the decreased expression and redistribution of occludin in retinal endothelial cells [134], demonstrating the altered glial-endothelial cell interactions results in iBRB breakdown [134, 144].

The interactions between astrocytes and vascular cells were studied both in vivo and in vitro [14, 146, 147]. Hyperglycemia increased astrocytes to produce many inflammatory cytokines such as interleukin (IL)-1β and IL-6 to amplify the inflammation in retina [148]. In DR, Ang-2 induces retinal astrocyte apoptosis through binding to avβ5 integrin [149]. The effect of high glucose on iBRB, using triple co-culture in vitro model with retinal pericytes, astrocytes, and endothelial cells, demonstrated that high glucose exposure upregulated IL-1β and IL-6 expressions, while downregulated ZO-1 and VE-cadherin expressions, which caused BRB breakdown and increased permeability [146].

### Microglia-endothelial interaction results in the microvascular destabilization

Microglias function as the tissue macrophages in retina. Microglias preferentially contact the superficial vascular plexus and neurons in the inner retina. As the sensors of the retinal microenvironment, highly branched/ramified microglia form a well-organized territorial network, scanning the defined area of the retina with their long processes [20]. Microglias transform into reactive phagocytes in responding to various insults with an amoeboid morphology.

Microglia could regulate the retinal blood flow. One study showed that, 4 weeks after diabetes onset in streptozotocin-induced rat model, decreased retinal blood flow together with retinal capillary constriction, as well as the increased microglial-capillary association were detected within the superficial capillary plexus [150]. The microglial-mediated retinal capillary regulation was through the fractakine-CX3C chemokine receptor 1 (CX3CR1)-induced angiotensinogen upregulation in microglia, which is via the angiotensin II receptor type 1-dependent mechanism.

In experimental DR, early activation of microglia was found [151], which was also reported by us [97, 152]. In the diabetic rat, activated microglia increased in cell numbers and also enhanced migration to the outer retina [97]. In our recent finding, activated microglia closely contact the retinal capillaries, phagocytosed the retinal endothelial cells after penetration of the vascular basement membrane, which is due to the decreased Src/Akt/Cofilin pathway signaling in activated microglia [97].

Besides the direct interaction with endothelial cells, microglia also mediate non-cell-autonomous death of retinal ganglion cells by producing TNFα [153]. Microglial activation contributes to iBRB breakdown through increasing multiple pro-inflammatory cytokines, VEGF and ROS. For example, high glucose-induced microglial IL-6 production reduced occludin and ZO-1, and high glucose increased microglial VEGF production through the activation of signal transducer and activator of transcription 3 (STAT3) [154]. In our recent work, we showed that activated microglia released inflammatory factors, including TNFα, iNOS, and IL-1β, which cause the death of both pericyte and endothelial cells, thus resulting in the iBRB breakdown, through phosphoinositide 3-kinase (PI3K)/protein kinase B (Akt)/STAT3/nuclear factor-κB (NF-κB) signaling pathway [155]. As a proof-of-concept, intravitreal injection of fractalkine, an inhibitor of microglial activation, decreased the expressions of the inflammatory factors and intracellular ROS in experimental DR [152].

The cell–cell contacts play the pivotal roles for the normal functionality of iBRB and iNVU, and the maintenance of microvascular stability. The above-mentioned interactions are complex, which include not only the direct interaction, e.g., endothelial-endothelial contact, pericyte-endothelial interaction, Müller glia-vascular cells interaction, and astrocyte-vascular cells interaction, but also the indirect interaction, e.g., Müller glia-endothelial interaction and microglia-endothelial interaction, reflecting the complexity for cell–cell contacts among different cells comprising the microvascular unit. Among these complex interactions, altered cytokines and signaling pathways are involved to cause microvascular destabilization in DR and DME.

## Altered cytokines and signaling pathways involved in microvascular destabilization in DR

In DR, many factors, including hypoxia, abnormal growth factors, cytokines, increased inflammatory cytokines and ROS, are involved in microvascular destabilization [9, 67, 68]. Hypoxia stabilizes hypoxia-inducible factor (HIF)-1α, which dimers with HIF-1β and translocases to the nucleus, stimulating hypoxia-regulated gene expressions such as VEGF, PlGF, Ang-2, vascular endothelial protein tyrosine phosphatase (VE-PTP) [156, 157]. These factors are interconnected, play together and contribute to the microvascular destabilization. Among these factors, VEGF and its receptors are the main stimulus for vascular leakage and retinal neovascularization, while Ang-2 and VE-PTP, by inhibiting Tie2, sensitize the endothelial cells more responsive to VEGF [156], together contributing to the pathogenesis of DR. Several growth factors and activated pathways involved in microvascular destabilization are discussed below.

### VEGF/VEGFR pathways

The VEGF family comprises 5 glycoproteins, i.e., VEGF-A (typically, and hereafter, referred to as VEGF), VEGF-B, VEGF-C, VEGF-D, and PlGF. These family members bind to the corresponding receptors, i.e., VEGFR-1/Flt-1 (fms-like tyrosine kinase 1), VEGFR-2/KDR (kinase insert domain-containing receptor)/Flk-1 (fetal liver kinase 1) and VEGFR-3/Flt-4 (fms-like tyrosine kinase 4), and VEGFR co-receptors neuropilin-1 and -2 (Nrp-1 and Nrp-2) [158, 159]. VEGF-A signals mainly through VEGFR-1 and VEGFR-2, which are abundantly expressed in the endothelial cells [160, 161], with VEGFR-2 being the main receptor for VEGF-A signaling [162]. VEGF-A promotes endothelial cell survival, migration and proliferation by activating the PI3K and MAPK pathways through binding to VEGFR-1/2. VEGF-B and PlGF only binds VEGFR-1 and activates the PI3K pathway to support endothelial cell survival and migration. While VEGF-C and VEGF-D binds VEGFR-3 and promotes lymphangiogenesis [163, 164]. VEGF/VEGFR signaling pathways induce the vascular permeability via disrupting the tight junctions and increasing transcellular permeability and induce angiogenesis through promoting endothelial cell survival and proliferation.

#### VEGF

In retina, VEGF is produced by many retinal cells, including Müller glia, astrocytes and microglia [66, 162], which is mainly hypoxia-dependent [165]. VEGF induced vascular permeability through increasing paracellular and transcellular transports [166]. The increased paracellular permeability is mainly mediated by the nuclear translocation of β-catenin, which increased the expression of the receptor for urokinase plasminogen activator (uPAR) and promoted pericellular proteolysis, while the increased transcellular transports is mediated by the caveolin-coated vesicles, requiring the activity of nitric oxide synthase 3 (NOS3) and the nuclear translocation of NOS3 and VEGFR-2 [83, 166, 167].

VEGF increases the paracellular transport mainly by destabilizing the endothelial cell junctions, i.e., decreasing the expressions of tight junctional proteins (e.g. occludin, claudins, ZO-1) and adherens junctions (e.g., VE-cadherin, and β-catenin), as well as increasing the phosphorylation of several junctional proteins [77] [168]. VEGF results in occludin phosphorylation through PKCβ activation [169, 170], downregulates occludin expression by upregulating free cytosolic β-catenin and upregulating uPAR [167], promotes the internalization and ubiquitination of occludin through Src-family kinases [171], enhances the endocytosis of VE-cadherin mediated by Rac activation/β-arrestin-2 [76], and disrupts VE-cadherin through increasing transglutaminase-2 activity [172].

Besides, VEGF increased the transcellular transport by increasing caveolae formation and the expressions of Cav-1 and PLVAP [13, 173]. VEGF upregulates ICAM-1 expression which mediates the leukocyte adhesions and leukostasis [174], increases vascular permeability via an endothelial NO synthase (eNOS)-dependent mechanism of transcytosis in caveolae [83], and induces PLVAP formation [84].

#### VEGF-B

The biologic function of VEGF-B in DR remains debatable. VEGF-B has two isoforms, i.e., VEGF-B167 and VEGF-B186 [175, 176]. VEGF-B167 is about 4 times more abundant than VEGF-B186, serving as the predominant isoform. VEGF-B167 can bind to pericellular heparin-like glycosaminoglycans and anchor it to ECM, through its C-terminal heparin-binding domain [175]. As for the angiogenic effects of VEGF-B, conflicting results were reported, e.g., some studies reported VEGF-B is angiogenic [177–179], while others not [180–182], in normal and pathologic conditions. For example, VEGF-B overexpression in mice caused pathological neovascularization in both retina and choroids and the breakdown of BRB [183]; while VEGF-B acts as the survival factor for vascular cells rather than an angiogenic factor by other study [184]. Targeting VEGF-B reduced the neovascularization in both retina and choroids in vivo [184], indicating VEGF-B could be served as the antiangiogenic target. Besides, VEGF-B is reported to have novel function, i.e., potent antioxidation by boosting the expression of key antioxidant enzymes [185].

The change of vitreous VEGF-B in patients with PDR were also reported with controversial results, as some showing no change of vitreous VEGF-B level [186], while others reported significantly increased [187, 188]. The positive correlation between elevated VEGF-B level and the increased central retinal thickness and macular volume in patients with DR indicates the contribution of VEGF-B to the microvascular destabilization [187, 188].

Given the above contradictory findings, the biological function of VEGF-B in retina remains enigmatic. The detailed roles of VEGF-B in different stages of DR as well as in DME merit further exploration.

#### PlGF

PlGF modulates vascular cell responses via binding to VEGFR-1. PlGF can amplify VEGF-A-induced effects through receptor crosstalk, which binds to VEGFR-1 and replaces VEGF-A to VEGFR-2 [159]. PlGF plays a major role to recruit inflammatory cells and promote the release of inflammatory cytokines [159]. Increased levels of PlGF were detected in aqueous, vitreous, and/or retina of patients with DR [159, 189, 190], which were correlated with severity of DR [159].

PlGF/VEGFR-1 activation cause vascular leakage, pericyte loss, angiogenesis, inflammation, and fibrosis [159, 191, 192]. PlGF/VEGFR-1 activation can lead to the degradation or decrease of ZO-1 and VE-cadherin [192, 193], causing the iBRB breakdown in DR. While PlGF knockout could increase the expressions of ZO-1 and VE-cadherin through activating Akt and inhibiting HIF1α/VEGF pathway, thus reducing diabetes-induced pericyte loss and acellular capillaries [192]. Intravitreal injection of anti-PlGF antibody (5D11D4) could significantly reduce the number of inflammatory cells (microglia and macrophages) in the retina in streptozotocin-induced diabetic mouse model [191]. Intravitreal injection of anti-PlGF antibody reduced the reactive gliosis in streptozotocin-induced diabetic mice [159] and decreased the fibrosis in laser-induced mice model [191].

In a 12-month clinical trial [194], the patients with high-risk PDR and DME were treated with laser photocoagulation alone or the combined treatments of laser photocoagulation and intravitreal injections of aflibercept, the fusion protein with high potency binding VEGF-A, VEGF-B and PlGF. The results showed the combined treatment can effectively improve the vision, reduce the edema, regress the microaneurysm and neovascularization, reduce the hard exudates, as well as decrease the aqueous VEGF, monocyte chemotactic protein (MCP-1), and GFAP levels [194].

### Ang pathways and VE-PTP

The Ang/Tie-2 system is essential for vascular stabilization, which plays a vital role in maintenance of the microvascular stability. The Ang family comprises four members (Ang-1, Ang-2, Ang-3, and Ang-4), in which Ang-1 and Ang-2 are widely characterized, while Ang-3 and Ang-4 are less studied [123, 195, 196]. In quiescent resting endothelial cells, Ang-1 is basally expressed and released extracellularly to maintain the vascular stability, while Ang-2 is synthesized and stored in Weibel-Palade bodies (WPB) by endothelial cells. Once the endothelial cells become activation, WPB-stored Ang-2 was released rapidly, accompanied with the increased Ang-2 transcription.

Both Ang-1 and Ang-2 binds to the membrane bound type I tyrosine kinase receptors, Tie1 and Tie2, with opposite effect. Ang-1 activates Tie2 as the main agonist, whereas Ang-2 inactivates Ties as the antagonist. Both Tie1 and Tie2 are primarily expressed in the endothelial cells, with Tie2 also expressed in pericytes [123, 195, 197]. Tie1 is the orphan receptor modulating the surface presentation and activation of Tie2 [198]. Normally, Tie1, directly interacting with Tie2 with its ectodomain, promotes Ang-induced vascular responses; but, in acute endotoxemia, the Tie1 ectodomain is rapidly cleaved, contributing to the loss of Ang2 agonist activity and resulting in the microvascular destabilization [198]. Beside Tie2 receptor, Ang-2 can also bind to integrin receptor to aggravate vascular destabilization [199, 200].

To modulate the activity of Tie2, VE-PTP, a receptor-type protein tyrosine phosphatase, also plays a pivotal role in vascular stability. VE-PTP is predominantly expressed in vascular endothelial cells, and is an important regulator of endothelial junction integrity and vascular permeability [201].

#### Ang-1/Tie2 signaling maintains microvascular stabilization

Under normal condition, Ang-1 is basally expressed by perivascular cells including pericytes with relatively consistent concentration, activating its downstream signaling that promotes the survival of endothelial cells and microvascular stability via Ang-1/Tie2 signaling and its several downstream pathways [123]. Ang-1/Tie2 signaling activation can (1) induce the expressions of eNOS and survivin, which lead to the survival of endothelial cells, by activating PI3K/Akt pathway; (2) inhibit gene transcriptions, including Ang-2, by phosphorylating the forkhead box O1 (FOXO1) and preventing its nuclear translocation; (3) suppress the expressions of the inflammatory genes, e.g., ICAM-1, vascular cellular adhesion molecule-1 (VCAM-1) and E-selectin, by inhibiting NF-κB through activating A20-binding inhibitor of NF-κB (ABIN2); and (4) cause cortical actin cytoskeleton stabilization by activating GTPase pathways (Rac1/Rap1 or Iqgap1/Rap1) [123]. As a proof-of-concept, intravitreal injection of recombinant modified Ang-1 protein restored the hierarchical vasculature and reduced the edema and hemorrhage in the retina in the mice model of the PDGFRβ mAb (ABP5)-induced absence of pericytes [202].

#### Ang-2/Tie2 signaling destabilizes microvasculature

Ang-2/Tie2 destabilizes the resting endothelial cells and primes them to respond to exogenous cytokines. Under pathological conditions, Ang-2 expression is increased, which, acting in a context-dependent manner, results in the microvascular destabilization and sensitizes the endothelial cells to VEGF-A stimulation [123].

Ang-2/Tie2 signaling can result in pericyte detachment/loss and pericyte death [197, 203], priming the retinal endothelial cells to respond to VEGF and other inflammatory factors through several mechanisms, including activation of FOXO1 target genes, Tie1 ectodomain cleavage, and Tie2 suppression [123]. Ang-2 induces pericyte dropout by proteolytic degradation of the vascular basement membrane and induces pericyte migration, highlighting the importance of Ang-2 in DR [204]. Increased levels of Ang-2 would destabilize the vessels by disrupting the tight junctions and causing the dropout of the vascular cells. For example, Ang-2 can reduce VE-cadherin level by phosphorylation [81]; induce the internalization and degradation of avβ3 integrin in endothelial cells through Ang-2/Tie2/integrin complex formation, which subsequently induces the phosphorylation of focal adhesion kinase (FAK) in the FAT domain at Ser910 [205]. Besides, a bi-directional reciprocal model was proposed, Ang-1/2 activates and controls pericyte function via Tie2-induced Calpain, Akt and FOXO3A signaling cascades in pericyte [197].

In diabetic rat retinas, Ang-2 expression was increased significantly (about 30-fold and more); and the pericyte loss was detected in the retinas [206]. A direct Tie2 agonist, Tie2.1-hexamer, was reported to stabilize the retinal vessel both in vitro and in vivo [207]. Ang-2 was increased in the intraocular samples of the patients with DR and other retinal or choroidal vascular diseases [208, 209]. The phase III, RCT studies (YOSEMITE and RHINE) showed that intravitreal injection of faricimab, a bispecific antibody binding both Ang-2 and VEGF-A, achieved robust vision gains and anatomical improvements in DME patients [210].

#### Ang-2/integrin pathway disrupts iBRB

Beside binding Tie2 receptor, Ang-2 can also binds to integrin receptor [123]. The effects of Ang-2 signaling via integrins in endothelial cells is context-dependent. In Tie2-negative tip cells, Ang-2 binds to and activates integrins (αvβ3, αvβ5, and α5β1), which induce FAK phosphorylation at Tyr397, activation of Rac1, and the migration of endothelial cells [200]; while in Tie2-positive stalk cells, Ang-2 binds Tie2, resulting in integrin degradation [200, 205]. As for the iBRB breakdown and microvascular destabilization, Ang-2 can destabilize the endothelial cells by directly activating α5β1 integrin, causing the actin cytoskeleton rearrangement and decreasing VE-cadherin in the junctions of endothelial cells [199]; result in pericyte loss or death through binding to α3β1 integrin receptor [124]; and result in astrocyte loss through binding to avβ5 integrin [149].

#### VE-PTP inactivates Tie2 further destabilizing the microvasculature

As mentioned above, the Tie2 signaling pathway plays an important role to maintain vascular homeostasis and stability [123]. In endothelial cells, VE-PTP is physically in close contact with Tie2 and inhibits Tie2 activation by dephosphorylation on Tie2. Inhibition of VE-PTP can induce tyrosine phosphorylation of FGD (FYVE, RhoGEF, and PH domain containing) 5, a GTPase exchange factor for cell division cycle 42 (Cdc42), and stimulate its translocation to the cell contacts to stabilize the endothelial junctions [211].

VE-PTP is increased under hypoxia in endothelial cells both in vitro and in vivo, and deactivates Tie2 by dephosphorating Tie2 [212]. Targeting VE-PTP by AKB-9778, a selective small-molecule inhibitor of VE-PTP, was demonstrated to stabilize the retinal and choroidal vessels both in vitro and in vivo by activating Tie2 and its downstream signaling through promoting Tie2 phosphorylation, and thus reduce vascular leakage and suppress the retinal and choroidal neovascularization [212].

In an open-label, dose-escalation clinical trial with 4 dose cohorts of 6 DME patients, AKB-9778, via subcutaneous injection, was well tolerated; the doses of 15 mg or more twice daily decreased macular edema and increased vision in some patients [213], showing efficacy of a VE-PTP inhibitor in stabilizing the retinal vessels in DME. Combo therapy of AKB-9778 and anti-VEGF (ranibizumab) decreased the central subfield thickness much more than those treated with ranibizumab alone in DME patients [214].

### PDGF-B/PDGFRβ pathway

The PDGF-B/PDGFRβ signaling is essential for vessel maturation and stabilization. Vascular maturation and stabilization involves the ECM deposition and pericyte recruitment [117]. PDGF-B, secreted by endothelial cells, acts as the pericyte chemokine; PDGFRβ is expressed by pericytes. The endothelial cells secreted PDGF-B as a homodimer (PDGF-BB). PDGF-B binds to heparin sulfate through its C-terminal “retention” motif [117, 215]. PDGF-B, binding to PDGFRβ, stimulates the migration and proliferation of pericyte, promoting the maturation and stabilization of the microvasculature [216].

In mouse model, the endothelial-pericyte dissociations and BRB breakdown was observed in adult mouse retina by using the anti-PDGFRβ antibody to transiently inhibit pericyte recruitment to the developing retinal vessels [116]. This phenotype can mimic several features of DR including increased vascular permeability, decreased perfusion, and neovascularization [116]. In this model, pericyte depletion induced the inflammatory responses of endothelial cells and increased the macrophages infiltration. At the leaky aneurysms, increased Ang-2, together with decreased Tie1, activated FOXO1 in local endothelial cells without pericyte coverage. This vicious cycle causing vascular damage can be inhibited by simultaneously targeting VEGF, PlGF, and Ang-2 [116].

Genetic deletion of PDGF-B or its “retention” motif in endothelial cells showed severe vascular impairments, including vascular engorgement and hyperpermeability, improper pericyte recruitment and coverage during the retinal vascular development [6, 217], which are highly mimicked by the PDGFRβ blocking antibody [202].

These studies indicated that endothelial cell derived PDGF-B can facilitate pericyte recruitment and maintain the microvascular stabilization during vascular development.

### TGF-β family

The TGF-β superfamily, consisting of more than 30 members, can be classified into different subfamilies, including TGF-βs (TGF-β1, TGF-β2, and TGF-β3), bone morphogenetic proteins (BMPs, about 20 members), growth differentiation factors (GDFs), nodals, and activins [218], and they play essential roles in many aspects, such as embryonic development, tumorigenesis, and inflammatory responses [219, 220]. In a large cohort of type 2 diabetic patients, plasma levels of growth differentiation factor 15 (GDF-15) were significantly higher in patients with DR compared to patients without DR [68]. The circulating GDF-15 concentrations were positively associated with DR progression after controlling the confounding risk factors [68].

#### TGF-β

TGF-β exerts its effect through binding to their cell surface transmembrane receptor serine/threonine kinases, including type I receptors activin receptor-like kinases (ALKs 1–7) and type II receptors TGF-β receptor 2 (TGFBR2) and BMP receptor 2 (BMPR2) [218]. Upon ligand binding, TGF-β transduces its signals through the canonical Smad-dependent (Smad 1/5/8 and 2/3), or Smad-independent (such as the MAPKs and PI3K/Akt) pathways [221] [222]. In the canonical signaling pathway, TGF-β-induced ALK5 activation phosphorylates Smad 2/3, which translocases to the nucleus and regulates gene expressions [218].

Under normal conditions, TGF-β is latent in both endothelial cells and pericytes, and regulates microvascular stability through promoting pericyte differentiation [101]. But under pathological conditions, TGF-β exerts the functions mediating angiogenesis, inflammation, and fibrosis [101]. TGF-β was detected to be highly increased in the vitreous of PDR patients [223, 224]. TGF-β, upregulated in Müller glia under hypoxia, can increase VEGF expression [225, 226]. TGF-β promotes occludin degradation via MMP-9 production, thus decreasing the iBRB [225]; and causes pericyte loss/apoptosis mediated by TGF-β-induced BIGH3 protein [129]. Deletion of TGF-β reduces the pericyte coverage, aggravating the vascular leakage as seen in DR [101]. Systemic deletion of PDGFRβ in pericytes downregulates TGF-β, forming the vicious cycle in microvascular destabilization [101]. In brain, endothelial cells regulate N-cadherin expression, a key adhesion protein for the pericyte-endothelial contract, through TGF-β/Smad4 and Notch pathways to maintain cerebrovascular integrity [227].

#### BMP

BMP family members are classified into several subgroups, such as the BMP2/4 group, the BMP5/6/7/8 group, the BMP9/10 group and the BMP12/13/14 group, and etc., based on their structural homology [228]. BMPs can bind to two types of serine-threonine kinase BMP receptors (BMPR1 and BMPR2), with a higher affinity for BMPR1 than BMPR2 [221, 228]. Upon ligand binding, BMPs transduce the signaling via either the canonical Smad-dependent pathway or the non-canonical pathways, such as MAPK, PI3K/Akt [222] [221, 228]. The increased levels of BMP2 and BMP4 were detected in retinas or vitreous of human and experimental DR [229, 230].

BMP2 promotes the microvascular dysfunction in DR via enhancing both pro-angiogenic and inflammatory pathways [230]. There exists a cross-talk between VEGF and BMP2. Studies showed that hypoxia- or VEGF-treated microvascular endothelial cells increased the expression of BMP2 [231]. The in vitro study showed BMP2 could induce VEGF secretion by Müller cells, causing the increased permeability of cultured human retinal endothelial cells; it can also induce leukocyte adhesion to the human retinal endothelial cells, upregulating the expressions of ICAM-1, IL-6 and IL-8 [230]. Besides the canonical Smad signaling, BMP2 can induce non-canonical inflammatory pathway in human retinal endothelial cells via activating p38 MAPK/NF-κB pathway, which increased VEGF expression and disrupted the barrier function of human retinal endothelial cells [232].

BMP3 is reported to regulate the blood–brain barrier integrity via enhancing pericyte development in zebrafish brain, since knockdown of BMP3 by morpholino oligonucleotide causes intracerebral hemorrhage and impairs the coverage of cerebral pericyte in zebrafish embryos [233]. However, its effect in DR, especially in the integrity of iBRB and the mammalian retina needs further exploration.

BMP4 was found to be upregulated in high glucose-treated vascular endothelial cells, and BMP4 could increase the expressions of Smad9, VEGF and fibrosis-related factors in vivo and in vitro [234]. BMP4 disrupted the tight junctions (ZO-1) of human retinal endothelial cells and impaired its barrier function (the transcellular electrical resistance) by increasing the expressions of phospho-Smad 1/5/9 and phospho-p38 [229]. Downregulation of BMP4 by shRNA was reported to inhibit angiogenesis and promote the apoptosis of retinal endothelial cell in experimental DR both in vivo and in vitro by decreasing both p-Smad1/5 and VEGF [235].

BMP9/ALK1 signaling was shown to prevent vascular dysfunctions in diabetic models, and adenoviral-delivered BMP9 expression reduced retinal vascular leakage and maintained the endothelial junctions in diabetic mice [236].

### PKC family

PKC family is a family with multifunctional serine/threonine kinases, which controls various protein functions. PKC activation induced by high glucose is associated with many pathological processes, such as retinal hemodynamics, vascular leakage, leukocyte adhesion and leukostasis, and increased VEGF expression [237]. PKC isoforms (at least 12 members) are subdivided into three groups, i.e., classical (α, β1, β2, and γ), novel (δ, θ, η, and ε), and atypical (ζ and ι/λ) groups. Chronic hyperglycemia activates PKC isoforms. Among PKC isoforms, PKCβ and PKCδ are preferentially activated in retinal vessels in diabetic animals.

Activation of the PKC pathway could cause vasoconstriction and decrease retinal blood flow. In endothelial cells, PKCβ isoform activation increases the expression of endothelin-1 and enhances VEGF function, resulting in the endothelial dysfunction and decreased retinal blood flow [238]. In the multicenter, double-masked, randomized, clinical trials, oral administration of PKCβ selective inhibitor (ruboxistaurin) was well tolerated and delayed vision loss and DME progression [239].

In retinal pericyte, hyperglycemia can increase the expression of PKCδ and activate p38α MAPK (PKCδ/p38α MAPK), which resulted in pericyte apoptosis and acellular capillaries via NF-κB and SHP-1/PDGFRβ pathways in vitro and in vivo [127]. Activated SHP-1 inactivates PDGFRβ and causes pericyte apoptosis by dephosphorylating PDGFRβ and decreasing its downstream signaling in pericyte [127].

### Semaphorin 4D (Sema4D)/PlexinB1 pathway

Semaphorins and their receptors (Plexins) play important roles in many vascular pathophysiological processes [240, 241]. Sema4D, also known as CD100, belongs to transmembrane semaphorin class IV. Sema4D can be proteolytic cleaved into its soluble form (sSema4D) and then shed from the cell surface. The level of sSema4D is significantly upregulated in the vitreous humor of patients with DR comparing to that in patients with macular hole, as well as upregulated in streptozotocin-induced mouse DR model [242]. The aqueous sSema4D, a strongly pro-angiogenesis and permeable factor, was reported to be positively correlated with the central subfield thickness in patients with DR [242]. The increased sSema4D in the aqueous of patients with DR was negatively correlated with changes in the central subfield thickness and macular volume with anti-VEGF therapy [243]. The increased Sema4D/PlexinB1 signaling could increase endothelial cell migration and vascular leakage, and also promote the N-cadherin internalization and pericyte loss, leading to vascular destabilization in DR [243]. Sema4D/PlexinB1 induced endothelial cell dysfunction via mammalian diaphanous homolog1 (mDIA1), which was mediated through Src-dependent VE-cadherin dysfunction under diabetic conditions [243].

The inhibition of Sema4D/PlexinB1 signaling mitigates vascular dysfunction in DR [243]. Besides, Sema4D knockout reduces the pathological retinal neovascularization in the oxygen-induced retinopathy mouse model and also reduces the vascular leakage in streptozotocin-induced mouse DR model [242]. A recent study using the smart supramolecular peptides (SSPs) to capture the sSema4D and downregulate its expression demonstrated that SSPs could be noninvasively and effectively transferred into vitreous humor of the mouse in the form of eye drop [242]. By encapsulating sSema4D, SSPs could reduce the pathological retinal neovascularization and vascular leakage, with synergistic effect when combining anti-VEGF treatment, in both the oxygen-induced retinopathy mouse model and streptozotocin-induced mouse DR model through inhibition of Sema4D/PlexinB1 signal pathway [242]. The clinical application of SSPs in patients with DR and other fundus vascular diseases merits further studies through investigator-initiated trial (IIT) and randomized clinical trial (RCT).

### S1P signaling pathway

Sphingolipids have been regarded as the important components of lipids, which are involved in several aspects, including signal transduction, cell proliferation, apoptosis, and membrane structure with pivotal roles. S1P is produced by phosphorylation of sphingosine via sphingosine kinase (SphK) types 1 and 2 (SphK1 and SphK2) [1]. S1P mediates many pathophysiological processes, controlling cell growth, differentiation, survival and death, through bound to G protein-coupled S1P receptors (S1PR1-S1PR5) [244]. S1PR1 signaling reduces the angiogenic sprouting by inhibiting VEGF-A-induced signaling and promotes cell-to-cell adhesion by stabilizing VE-cadherin localization at the junctions of endothelial cells [245]. S1P could stabilize the vessels by activating endothelial S1PR1 in both development and homeostasis [246]. S1P, derived from pericyte, increases the expressions of N-cadherin and VE-cadherin within pericyte-endothelial cell contact and reduces Ang-2 in endothelial cells, increasing the integrity of endothelial cells and reducing angiogenesis [247]. S1PR2 was upregulated in pericytes in ischemia, which reduced N-cadherin expression and promoted the migration of pericytes via NF-κB p65 signaling, causing the breakdown of blood–brain barrier [248]. In retina, S1PR2 signaling disrupted the endothelial junctions, stimulated VEGF expression and release, and increased retinal neovascularization, resulting in the vascular destabilization [247]. In one prospective cross-sectional study, the serum level of S1P was found to be higher in individuals with DR compared to healthy individuals; and the aqueous level of S1P was significantly higher in patients with PDR than others with NPDR or non-DR [249].

Due to the complex roles of S1P and its different receptor subtypes in angiogenesis, inflammation, apoptosis and fibrosis, the changes of S1P and its receptor subtypes should be determined before targeting S1P to treat ocular vascular diseases [247]. In the mouse model of oxygen-induced retinopathy, endothelial overexpression of S1PR1 suppresses neovascular tuft formation; while in mice constitutively overexpressing ApoM (Apom^TG^) with more circulating HDL-S1P, circulating HDL-S1P could activate the endothelial S1PR1 and suppress the neovascular pathology [250]. Besides, systemic administration of ApoM-Fc-bound S1P or a small-molecule Gi-biased S1PR1 agonist was found to reduce neovascular tuft formation in the mouse model of oxygen-induced retinopathy [250], indicating the activation of endothelial S1PR1 could serve as a potential protective mechanism to suppress the neovascular retinopathy, such as retinopathy of premature and PDR.

### Ephrin-B2

The erythropoietin-producing human hepatocellular (Eph) receptors and their membrane-tethered ligands, the ephrins, play roles in mediating the communication between endothelial cells and pericytes [251]. Ephrin-B2, a tyrosine kinase regulating pericyte-endothelial cells communication during angiogenesis, is important for pericytes and endothelial cells to assemble into a vascular structure [252]. Ephrin-B2 and its receptor Eph-B4 was found to be upregulated in patients with PDR, indicating ephrin-B2 is a key regulator of neovascularization [253, 254]. Ephrin-B2, locating on the pericytes, and its receptor Eph-B4, locating on the endothelial cells, contributes to physiological and pathological angiogenesis [255].

Hyperglycemia increased Ephrin-B2 expression in the pericytes, which contributed to diabetes-induced retinal inflammation and vascular cell death, and its silencing reduced the pathological angiogenesis and formation of acellular capillaries in diabetic retinas [256]. Ephrin-B2 can regulate the internalization of PDGFRβ in pericytes [257], its down-regulation resulted in PDGFRβ redistribution from caveolin-positive membrane to clathrin-associated membrane fractions in cultured vascular smooth muscle cells, which promoted PDGF-B-induced PDGFRβ internalization and activated MAPK and c-Jun N-terminal kinase (JNK) pathways [257].

The microvascular alterations and destabilization in DR suggest the roles of the aberrantly expressed growth factors, possibly acting in combination, to drive this pathological process. As discussed above, multiple cytokines and signaling pathways are altered in DR and DME, which synergistically leads to microvascular destabilization, and thus the pathogenesis of DR.

### Intricated network of the cytokines for microvascular destabilization

The roles of different kinds of cytokines, including VEGF, PlGF, PDGF-B, Ang-1/2, TGF-β, and etc., are intimately linked via autoregulatory loops that coordinately maintain the microvascular stability in normal retina. However, in DR, these cytokines interact in networks and produce the opposite effect, resulting in microvascular destabilization and leading to vascular leakage, pericyte dropout, and even sprouting angiogenesis (Fig. 5).

Under normal condition, the survival of endothelial cells is supported by VEGF through binding to VEGFR-1. The microvascular stabilization is maintained together by endothelial cells-derived PDGF-B, which recruits the coverage of the pericytes, and by the pericytes-derived Ang-1 and TGF-β through binding to Tie2 and TGF-β receptors [258]. PDGF-B increases Ang-1 expression, and also the ratio of Ang-1/Ang-2, thus promoting the microvascular stabilization; PDGF-B can also increase TGF-β expression, promoting pericyte differentiation. Ang-1 activates Tie2 in endothelial cells to maintain the microvascular stability. The activation of Tie2 leads to activates PI3K/Akt pathway, phosphorylates FOXO1 and inhibits its nuclear translocation, inhibits NF-κB and suppresses the expressions of the inflammatory factors, as well as activates GTPase pathways in endothelial cells.

In DR, hypoxia and hyperglycemia increased the expressions of VEGF, PlGF, Ang-2, and VE-PTP, and etc. The increased VEGF and PlGF activate the endothelial cells, break down the iBRB, and promote the inflammation through binding to VEGFR-1/2. The activated endothelial cells produce and secrete amount of Ang-2, which results in the pericyte and astrocyte loss through binding to integrin, sensitizing the endothelial cells to VEGF. Increased Ang-2 destabilizes the endothelial cells through binding to Tie2 or integrin, leading to the vascular leakage, inflammation, and even neovascularization. VE-PTP dephosphorylates Tie2 and inactivates Tie2. The involvement of inflammation also aggravates the microvascular destabilization. The activation of nuclear factor of activated T cells (NFAT) in endothelial cells upregulates the expressions of inflammatory chemokines and leukocyte adhesion molecules, attracting the circulating monocytes to infiltrate the retinas. These inflammatory cells, e.g., macrophages and the endogenous microglia, secrete VEGF, PlGF, TNFα, and other proinflammatory factors, further amplifying the inflammation. The VEGF/VEGFR-2 signaling further activates NFAT and increase Ang-2 expression. Macrophages-derived VEGF and TNFα facilitates the cleavage of Tie1 ectodomain and the conversion of Ang-2 from the Tie2 agonist to the antagonist. This positive feedback loop sensitizes endothelial cells to the stimulus of VEGF and TNF-α, and sustains inflammation and vascular dysfunction. These finally result in the microvascular destabilization, leading to the breakdown of iBRB with increased paracellular and transcellular leakage.

## Insights into microvascular destabilization in DR

Recent advances have led to the emergence of the concept that DR is a disorder of retinal NVU [8], emphasizing the intimate relationship among the retinal neurons (photoreceptors, bipolar cells, ganglion cells, horizontal cells and amacrine cells), glial cells (Müller glias, astrocytes and microglias), vascular cells (endothelial cells and pericytes), RPE cells, the immune cells (microglia and perivascular macrophages), as well as the ECM (endothelial glycocalyx and vascular basement membrane). Coordinating the proper functions of all elements in NVU is essential and of great importance for the neural retina to adapt to various physiological conditions, i.e., the functional hyperemia. In iNVU, retinal endothelial cells via PDGF-B signaling recruit pericytes, which promote the barrier properties of the endothelium to maintain the integrity of iBRB [259]. Besides, Müller glias and astrocytes, maintains iBRB through providing the Norrin signaling and other molecules.

In DR, all the components of NVU are affected. Hyperglycemia can cause the loss of endothelial glycocalyx, the increased thickness of vascular basement membrane, the dysfunction and cell death of the endothelial cells, and the loss or dropout of pericytes, which lead to the microvascular destabilization of the retinal vascular unit, manifesting the breakdown of iBRB (vascular leakage), and the formation of microaneurysm (vascular leakage) and acellular capillaries (non-perfusion). Pericyte dropout results in the hyper-responsiveness of endothelial cells to VEGF signaling, which promotes the proliferation and migration of endothelial cells, eventually leading to the retinal neovascularization. In DR, glial cells react differently and cause the microvascular destabilization. Müller glia and astrocytes produced a large amount of vasoactive substances, such as VEGF-A, Delta-like protein 4 (Dll4), angiopoietin-related protein 4 (ANGPTL4), and leucine-rich α2-glycoprotein 1 (LRG1), promoting the vascular permeability and angiogenesis in retina [259]. Activated microglias increase the production of inflammatory cytokines, such as TNF-α, IL-1β, and MCP-1, aggravating the disease severity.

Besides, multiple molecules and signaling pathways are altered in DR. These pathways include but not limited to VEGF/VEGFR pathways, Ang pathways and VE-PTP, PDGF-B/PDGFRβ pathway, TGF-β family, PKC family, Sema4D/PlexinB1 pathway, S1P signaling pathway, and Ephrin-B2. These cytokines interact in networks and work synergistically, resulting in microvascular destabilization and further leading to vascular leakage, endothelial dysfunction, pericyte dropout, and even sprouting angiogenesis.

**Fig. 6:**
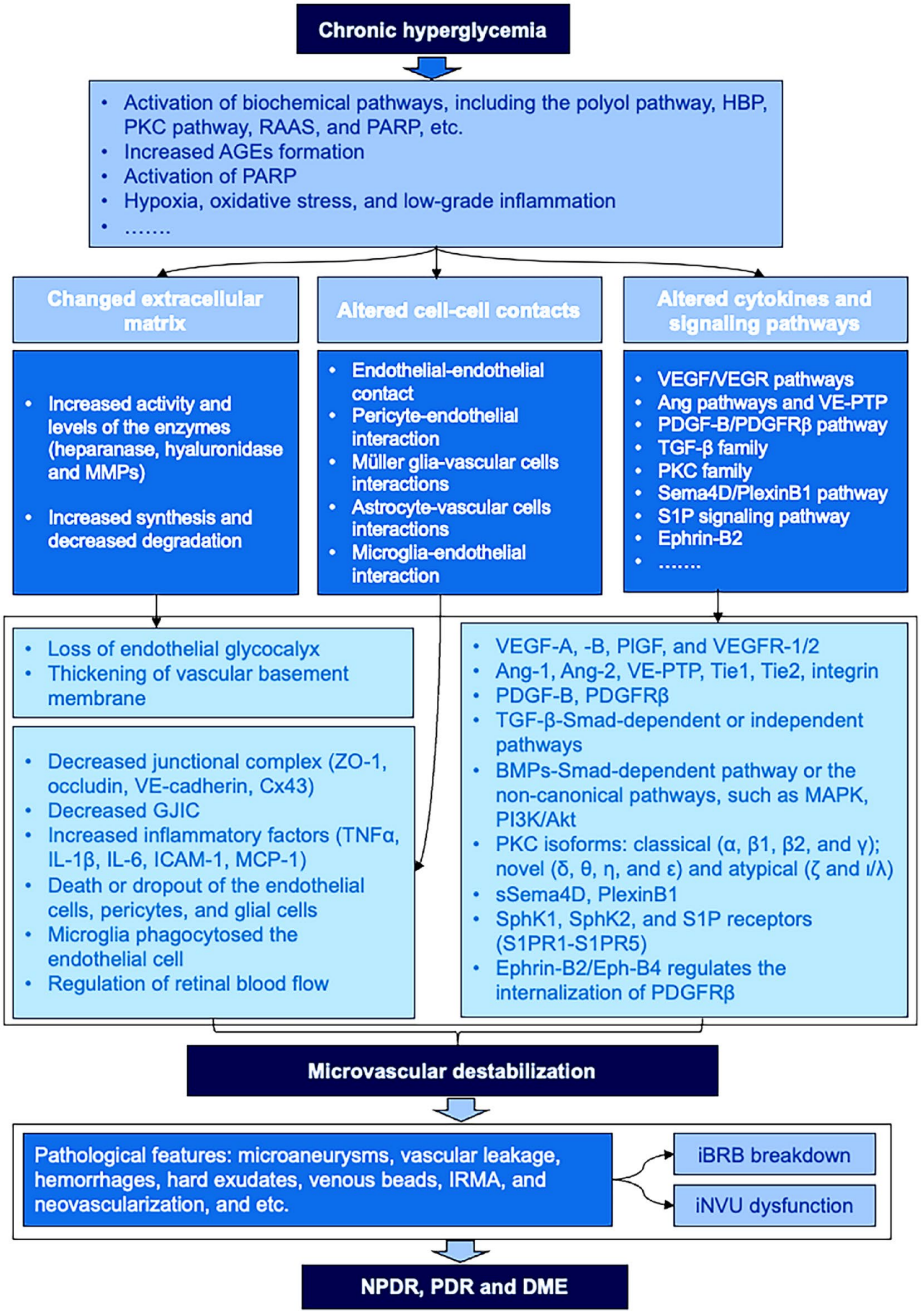
The comprehensive schematic diagram showing multiple factors are involved in the microvascular destabilization. The chronic hyperglycemia activates multiple biochemical pathways, including the polyol pathway, HBP, PKC pathway, increased AGEs formation, activation of RAAS, and etc. These activated biochemical pathways cause oxidative stress, low-grade inflammation, as well as activation of PARP in the retina. Multiple factors altered the noncellular components (extracellular matrix) of the retinal vascular unit, resulting in the loss of endothelial glycocalyx and thickening of vascular basement membrane. These factors can also alter the cell–cell contacts, including endothelial-endothelial contact, pericyte-endothelial interaction, Müller glia-vascular cells interaction, astrocyte-vascular cells interaction, and microglia-vascular cells interaction. Multiple pathways, including but not limited to VEGF/VEGFR pathways, Ang pathways and VE-PTP, PDGF-B/PDGFRβ pathway, TGF-β family, PKC family, Sema4D/PlexinB1 pathway, S1P signaling pathway, and Ephrin-B2, interact in networks and work synergistically, resulting in microvascular destabilization. The altered extracellular matrix and cell–cell contacts of the vascular unit as well as the increased multiple cytokines and grow factors together compromise the integrity and the function of the vascular unit, resulting in the breakdown of iBRB and the dysfunction of iNVU, further leading to vascular leakage, endothelial dysfunction, pericyte dropout, and even neovascularization in DR. AGEs: advanced glycation end-products; Akt: protein kinase B; Ang-1/2: angiopoietin 1/2; BMP2: bone morphogenetic protein 2; BMP4: bone morphogenetic protein 4; Cx43: connexin 43; DME: diabetic macular edema; DR: diabetic retinopathy; GJIC: gap junction intercellular communication; HBP: hexosamine biosynthetic pathway; iBRB: inner blood-retinal barrier; ICAM-1: intercellular adhesion molecule-1; IL-1β: interleukin-1β; IL-6: interleukin-6; iNVU: inner neurovascular unit; IRMA: intraretinal microvascular abnormalities; MAPK: mitogen-activated protein kinase; MCP-1: monocyte chemotactic protein 1; MMPs: matrix metalloproteinases; NPDR: non-proliferative diabetic retinopathy; PARP: Poly(ADP-ribose) polymerase; PDGF-B: platelet-derived growth factor; PDGFRβ: platelet-derived growth factor receptor β; PDR: proliferative diabetic retinopathy; PI3K: phosphoinositide 3-kinase; PlGF: placental growth factor; RAAS: renin–angiotensin–aldosterone system; S1P: sphingosine-1-phosphate; S1PR: sphingosine-1-phosphate receptor; Sema4D: semaphorin 4D; SphK: sphingosine kinase; Tie1/2: tyrosine kinase with immunoglobulin-like and the epidermal growth factor-like domains 1/2; TNFα: tumor necrosis factor α; VCAM-1: vascular cellular adhesion molecule-1; VEGF-A: vascular endothelial growth factor A; VEGFR-1/2: vascular endothelial growth factor-1/2; VE-PTP: vascular endothelial protein tyrosine phosphatase; ZO-1: zonula occludens-1

Due to the complex pathogenesis for the microvascular destabilization (Fig. 6), several questions remain to be discussed and merit further exploration. For example, for the cellular and noncellular components of the vascular unit, which initiates the disease process first after diabetic onset? do these components initiate the disease process independently or inter-dependently during diabetes progression? is there a common underlying molecular mechanism of the changes of the cellular and noncellular components of the vascular unit? Besides, among the multiple altered molecules, which cytokines or combinations of cytokines play the pivotal roles in triggering or transducing the disease process to cause the microvascular destabilization?

## Limitations and suggested areas for future research

This review has some limitations that deserve attention. The papers published up to June, 6, 2024 and written in English were included, which may cause possible publication bias. Other studies or groups that could contribute insight into vascular destabilization may have excluded the Internet use, which were ignored in this review.

The scope and coverage limitations were that most of the studies included were related to the pathophysiology of the retinal microvasculature, mainly focusing on DR and DME. Other vascular diseases including other neovascular ocular diseases, e.g., neovascular age-related macular degeneration, choroidal neovascularization secondary to pathologic myopia, and retinal vein occlusion, and the systemic vascular diseases, e.g., cerebrovascular diseases (stroke, transient ischemic attack, and intracranial vascular malformation, and etc.) and cardiovascular diseases (ischemic heart disease, hypertension and atherosclerosis, and etc.), should be also considered and discussed further, which merits the suggested areas for future research. Besides, the cellular interactions and intracellular communications, as well as the detailed molecular interplays involving the microvascular destabilization have not been fully discussed, which also merit future research.

## Conclusion and perspective

The breakdown of iBRB results in increased vascular permeability, retinal edema, and neuroretinal damage in iNVU, causing loss of vision in DR. The iBRB breakdown is due to the microvascular destabilization of the retinal vascular unit (Fig. 2), especially the retinal endothelial cells, which is caused by the combinational effects of increased levels of growth factors and cytokines, sustained inflammation, and dropout/loss/death of pericytes and endothelial cells due to hyperglycemia, hypoxia, or other insults (Fig. 6). Subsequently, both paracellular and transcellular transports increase across the retinal endothelium through the opening/disruption of endothelial intercellular junctional complexes and/or the altered endothelial caveolar transcellular transport. The functional and structural changes of the iBRB and the extracellular components (the composition of the endothelial glycocalyx and the ECM of iBRB) further facilitate and aggravate the breakdown of iBRB, and leading to the hyperpermeability, macular edema, and even neuroretinal damage and dysfunction in iNVU.

The iBRB breakdown and the dysfunction of iNVU is primarily due to the microvascular destabilization, which involves the complex cellular and molecular processes in DR (Figs. 5 and 6). The advancements understanding the microvascular destabilization in DR have grown rapidly in recent years. Targeting the key molecules or specific iBRB cells will provide the insights for rational design of drugs aimed at stabilizing the microvasculature in retina, thus restoring the function and structure of iBRB and iNVU, to treat DR and DME.

## Data Availability

The data are available from the corresponding author on reasonable request.
